# Aquaporin 1 is renoprotective in septic acute kidney injury by attenuating inflammation, apoptosis and fibrosis through inhibition of P53 expression

**DOI:** 10.3389/fimmu.2024.1443108

**Published:** 2024-08-22

**Authors:** Wuyang Lv, Jia Liao, Cuicui Li, Dongyang Liu, Xiaoxiao Luo, RuXue Diao, YuChen Wang, Yingyu Jin

**Affiliations:** ^1^ Department of Clinical Laboratory, the First Affiliated Hospital of Harbin Medical University, Harbin, Heilongjiang, China; ^2^ Department of Clinical Laboratory, Shangluo Central Hospital, Shangluo, Shaanxi, China

**Keywords:** sepsis, AKI, aquaporin 1, p53, inflammation, apoptosis

## Abstract

Sepsis associated Acute kidney injury (AKI) is a common clinical syndrome characterized by suddenly decreased in renal function and urinary volume. This study was designed to investigate the role of Aquaporin 1 (AQP1) and P53 in the development of sepsis-induced AKI and their potential regulatory mechanisms. Firstly, transcriptome sequencing analysis of mice kidney showed AQP1 expression was reduced and P53 expression was elevated in Cecal ligation and puncture (CLP)-induced AKI compared with controls. Bioinformatics confirmed that AQP1 expression was remarkably decreased and P53 expression was obviously elevated in renal tissues or peripheral blood of septic AKI patients. Moreover, we found *in vivo* experiments that AQP1 mRNA levels were dramatically decreased and P53 mRNA significantly increased following the increased expression of inflammation, apoptosis, fibrosis, NGAL and KIM-1 at various periods in septic AKI. Meanwhile, AQP1 and P53 protein levels increased significantly first and then decreased gradually in kidney tissue and serum of rats in different stages of septic AKI. Most importantly, *in vivo* and vitro experiments demonstrated that silencing of AQP1 greatly exacerbates renal or cellular injury by up-regulating P53 expression promoting inflammatory response, apoptosis and fibrosis. Overexpression of AQP1 prevented the elevation of inflammation, apoptosis and fibrosis by down-regulating P53 expression in Lipopolysaccharide (LPS)-induced AKI or HK-2 cells. Therefore, our results suggested that AQP1 plays a protective role in modulating AKI and can attenuate inflammatory response, apoptosis and fibrosis via downregulating P53 in septic AKI or LPS-induced HK-2cells. The pharmacological targeting of AQP1 mediated P53 expression might be identified as potential targets for the early treatment of septic AKI.

## Introduction

Sepsis is a refractory clinical syndrome characterized by systemic inflammation and multiple organ dysfunction caused by a patient’s dysregulated response to infection (1). The prevalence of septic AKI is increasing and the mortality can be as high as 50%∼80% (1). An increasing number of evidences suggests that the pathological mechanism of septic AKI is complex according to the difference of pathogenic factors (2). However, the exact pathologic mechanism in septic AKI is still unknown and absence of early diagnostic biomarker (1, 2). In addition, septic AKI is not detected and treated in a timely manner, which greatly increased the risk of AKI transition to Chronic kidney injury (CKD), reducing the prognostic effect and postnatal quality of patients and increasing the economic burden of patients (1, 2). Therefore, the mechanism of renal injury and repair following septic AKI or the process of AKI transition to CKD has become a hot topic in current studies.

AQP1, a member of the aquaporin family, is a transmembrane protein that transports water molecules with a molecular weight of 28kDa, and had a molecular weight of 36kDa if glycosylation was taken into account (3). The kidney is an important organ in maintaining water homeostasis in the body by concentrating and diluting urine (4). The kidney regulates water reabsorption through different AQPs, especially AQP1 (4). In the kidney, AQP1 is mainly expressed in the proximal tubule, thin descending limb of Henle and descending vasa recta (5). Our previous study found that AQP1 is involved in regulating the immune response in the kidneys of septic AKI. In experimental septic AKI rat model and LPS-induced RAW264.7 macrophages, AQP1 facilitated macrophages M2 polarization through PI3K or inhibition of P38 MAPK pathway, significantly attenuated renal inflammatory response, and thus promoted the recovery of injured tissues (6, 7). Moreover, our results in LPS-stimulated HK-2 cells showed that si-AQP1 knockdown of AQP1 in HK-2 cells further promoted the secretion of inflammatory factors and apoptosis, while overexpression of AQP1 significantly reversed LPS-induced cellular injury (8). Therefore, these experimental results suggest that AQP1 has renal protective effect on septic AKI.

P53, a well-known tumor suppressor protein, which are closely related to the pathogenesis of cancer (9). Since its discovery in 1979, P53 has been a focus of oncology research (9). Cellular stress responses, including DNA damage, oncogene expression, hypoxia, reactive oxygen species (ROS) and nutrient deprivation can induce upregulation of p53 expression. In recent decades, accumulating researches have demonstrated that P53 plays an important role in other diseases besides tumors (10). Investigators (11) reported that renal p53 expression was up-regulated observably in medulla within 24 h after ischemia/reperfusion (IR). However, PIF chemically inhibits p53 activity will be preventing tubular cell apoptosis and leading to reduced renal impairment during AKI (11). In 2010, researchers demonstrated that P53 regulates the G2/M phase of cells to promote cell cycle inhibition and aggravate renal fibrosis, suggesting that it plays a key role in the pathogenesis of CKD. Moreover, Baisantry et al (12) reported that p53-siRNA was continuously injected into rats 14 days in ischemia-reperfusion induced AKI, which significantly reduced aging load and injury phenotype after ischemia. These findings suggest that time dependent p53 inhibition determines senescence attenuation and long-term outcome after renal ischemia/reperfusion injury. The P21 is an important member of the family of cell cycle protein-dependent kinase inhibitors discovered in recent years (13). The discovery and cloning of the P21 and its important role in cell cycle control and tumorigenesis (13). Researches show that P21 was significantly upregulated prior to AKI and also serves as a hallmark of P53 transcriptional upregulation (13).

Interestingly, researchers have been reported in hypoxia-induced pulmonary hypertension that targeted knockout of AQP1 gene eliminated the proliferation and migration potential of pulmonary smooth muscle cells, increased the expression of apoptosis, and alleviated hypoxia-induced pulmonary hypertension (14, 15). Importantly, the researchers showed that this effect was associated with significantly upregulation of P53 expression after targeted AQP1 knockout (14, 15). Thus, these findings suggest that there may be a potential association between P53 and AQP1. Unfortunately, the researchers did not conduct further explore on the potential relationship network between AQP1 and P53.

In summary, previous investigations found that AQP1 and P53 have a potential relationship in hypoxia-induced pulmonary hypertension and are crucial to the occurrence and development of the disease. However, whether AQP1 regulates P53 expression and the underlying mechanisms remain unknown in septic AKI. Therefore, this study constructed septic AKI model in rats by intraperitoneal injection of LPS or CLP, mainly studying whether AQP1 influence renal damage and repair of septic AKI by regulating P53 expression.

## Materials and methods

### Reagents

Lipopolysaccharide (LPS, Escherichia coli serotype 0111: B4, L4130, Sigma Aldrich, St. Louis, MO, USA). Dulbecco’s modified Eagle’s medium (DMEM) (Gibco, Beijing, China), fetal bovine serum (FBS) (Procll Life Science&Technology Co.,Ltd, Wuhan, China), and penicillin–streptomycin (Solarbio, Beijing, China). Penicillin−streptomycin (CM-0129, Procell Life Science and Technology Co.,Ltd, Wuhan, China). Pentobarbital sodium (CAS number:57-33-0, Sigma-Aldrich, USA). The antibodies used in this study were as follows: Anti-AQP1 antibody (WL00730; Wanleibio, Jilin, China), Anti-P53 (WL01919; Wanleibio, Jilin, China), Anti-P21 (ab109520; Abcam, USA), Anti-CD68 (ab283654; Abcam, USA), Anti-β-actin (Zhongshan jinqiao Biological, China), a goat anti-Rabbit IgG (Proteintech, Hubei, China), a goat anti-mouse IgG (Abcam, China). Phosphatase inhibitors for 30 min (Biosharp, China). Chemiluminescence-plus reagents (WB100D, NCM Biotech). All rat enzyme−linked immunosorbent assay kits, including AQP1, P53, P21, IL-1β, IL-6, TNF-α and pNF-kB, were obtained Jianglai Biology (Shanghai, China). RNAiso Plus reagent (Code No.9109, Takara), Script cDNA Synthesis Kit (Code No. RR036A, Takara). TB Green Premix (Takara). Annexin V-FITC/PI Apoptosis Detection kit (BD Biosciences). Transfection reagent jetPRIME and jetPRIME buffer were purchased Polyplus(France), pcDNA3.1-AQP1 (V79020, Thermo Fisher Scientific, USA). RiboFECT™CP Transfection Kit and small interfering-AQP1 were purchased RiboBio (Guangzhou, China). PMSF (Solarboi life Sciences, Beijing, China). BCA Protein Assay kit (Solarboi life Sciences, Beijing, China). Pifithrin-α (HY-15484), Kevetrin hydrochloride (HY-16271) and Cell Counting Kit-8(CCK8, HY-K0301) assay kit were obtained from MedChemexpress (Monmouth Junction, USA).

### Acquisition of transcriptional sequencing data

Public microarray database GEO (Gene Expression Omnibus, http://www.ncbi.nlm.nih.gov/geo/) was searched for AKI-related samples. Raw data (Excel file) from GSE186822, GSE220812, GSE67401, GSE248078 and GSE232404 were downloaded from the GEO database, respectively. GSE186822 and GSE220812 derived total kidney mRNAs from mice with Cecal ligation and puncture (CLP)-induced AKI. GSE67401 derived peripheral blood RNA of 133 representative subjects with systemic inflammatory response syndrome that had AKI or Hemodialysis (HD). GSE248078 derived renal tissue RNA of 4 patients with postoperative Contrast-induced AKI (CI-AKI) and 4 patients without CI-AKI. GSE232404 derived 10 samples of whole blood RNA of adults, including 5 cases of septic AKI and 5 cases of healthy controls. The robust multiarray averaging method was then used to preprocess the raw data. Probes were annotated with the latest official annotations file. All bioinformatics analyses were processed and analyzed by RStudio and GraphPad Prism (8.0.1).

### Compliance with ethical considerations

The male Wistar rats (180-220 g, aged 8-9 weeks) were obtained from the Laboratory Animal Center of Harbin Medical University, Heilong jiang, China. This study protocol was reviewed and approved by the Harbin Medical University Institutional Animal Ethics Committee. Extensive efforts were made to minimize animal usage and suffering. All animals were housed in individual and standard cages under controlled environment with temperature control (23±2°C), and the rats had free access to water and food. LPS induced AKI models were used in present study.

### Experimental design

Two septic AKI models were established with LPS (10mg/kg body weight) via intraperitoneal injection (7) and Cecal ligation and puncture (CLP). Firstly, animals were randomly divided into two groups of twenty-one rats each. They were treated as follows: Control group: sterile saline solution (intraperitoneal injection), LPS group: LPS solution (intraperitoneal injection). Control group or LPS group was further divided into subgroups of (8 h, 12 h, 24 h, 48 h, 72 h, 7 d and 14 d) according to different points after fluid injection, with three animals in each group. Secondly, twenty-four rats were randomly assigned into one of the two experimental groups: AQP1 silencing group (n=12) and AQP1 overexpression group (n=12). According to the experimental grouping requirements: 1) the AQP1 silencing group consisted of Control group, LPS group, LPS+si-AQP1 group (rats were pre-injected intravenously by tail vein with 10 nmol of si-AQP1 every 24 h for 3 d, followed by injection of LPS), LPS+si-AQP1+PIF group (pre-tail vein injection of rat si-AQP1, intraperitoneal injection of PIF followed by LPS injection within 30 min). 2) AQP1 overexpression group mainly includes: Control group, LPS group, LPS+pcDNA3.1-AQP1 group (pre-tail vein injection of 0.7μg of pcDNA3.1-AQP1 in rats and injection of LPS after 72 h), LPS+pcDNA3.1-AQP1+Keve group (rats were injected with 0.7μg of pcDNA3.1-AQP1 in the tail vein and injected intraperitoneally with a Kevetrin hydrochloride after 72 h, and LPS was injected within 30 min). Pifithrin-α (PIF, 3mg/kg body weight) working solution: Pifithrin-α was dissolved in 1% DMSO and configured as mother solution, then physiological saline was added and heated or slightly shook in 37°C water bath to prepare Pifithrin-α working solution. Kevetrin hydrochloride (1.5mg/kg body weight) working solution: Kevetrin hydrochloride was dissolved in 2% DMSO and configured as mother solution, then PBS was added to prepare Kevetrin hydrochloride working solution.

Rats were anesthetized by intraperitoneal injection of 3% pentobarbital sodium solution (0.2 ml/100 g body weight). Blood was collected by cardiac puncture with a 5 ml sterile syringe, and the serum was separated by centrifugation at 3500 rpm for 10 min at room temperature and stored in separate packages at -80°C. Subsequently, kidneys were removed and washed with sterile saline for subsequent experiments.

A total of 6 male Wistar rats were randomly divided into 2 groups: CLP group (n=3), Sham group (n=3). The rats sepsis model was established with the classic CLP method (16). Briefly, rats were anesthetized by the intraperitoneal injection of 3% pentobartital sodium. The cecum was ligated below the ileocecal valve by using 4-0 silk ligatures. Then the cecum was perforated at 2 positions with a 21-gauge needle and gently compressed to extrude the feces. The guts were then returned to the abdomen and the incision was closed. For the sham rats, they were subjected to the same surgical procedures without ligating the puncturing the cecum. All rats received fluid resuscitation with saline solution (30 ml/kg) by subcutaneous injection after the surgery. At 24 h of CLP treatment, the rats were anesthetized by intraperitoneal injection of 3% pentobarbital sodium solution (0.2 ml/100 g body weight). Blood was collected by cardiac puncture with a 5 ml sterile syringe, and kidneys were taken for subsequent experiments.

### Assessment of renal function

Serum creatinine (SCr) and blood urea nitrogen (BUN) were analyzed by an automatic biochemical analyzer (Vitros V5600, Johnson, USA). A 1.5-fold increase in serum creatinine compared with the control group demonstrated that a successful establishment of the LPS induced AKI model (17).

### Histopathologic analyses

Rat kidney tissues were taken, washed in sterile saline and fixed with 4% paraformaldehyde for 24 h, then dehydrated with different concentrations of ethanol. Subsequently, paraffin-coated and sectioned into 4μm thickness, and subjected to HE staining and Masson staining, respectively. The staining results were observed under a microscope for image acquisition and analysis (Olympus BX40, Tokyo, Japan) to evaluate the extent of renal tubular damage and morphological changes as well as interstitial fibrosis. The renal injury was defined as loss of the tubule brush border, renal tubule swelling and renal interstitial fibrosis in the cortex (2, 18). Finally, statistical analysis and graphing were performed, according to the scoring results.

### Immunohistochemistry analysis and evaluation

Rat kidneys were fixed in 4% paraformaldehyde for 48 h and paraffin-embedded. Serial paraffin sections were baked in a 65° oven for 60 min to prevent the tissue from falling off the slides. Three vats of dewaxing solution (xylene) were used for 10 min each to remove the paraffin embedded in the kidney tissue. Four tanks of gradient alcohol (100%, 100%, 95%, 75%) were used for 2 min each, followed by a 2 min immersion in purified water. 3% hydrogen peroxide immersion for 5 min to destroy endogenous peroxidase on the tissue. The repair cassette was filled with EDTA repair solution, the sections were placed, the cassette was placed in a 100° water bath for 20 min, cooled down naturally and soaked in PBS for 2 min. The sections were blocked with serum for 1 h at room temperature, then incubated with primary antibody CD68 (1:300) and incubated at 4°C for 18 h. Sections were washed three times with PBS and incubated with secondary antibody (1:500) for 1 h at room temperature. The DAB working solution was compounded to cover the tissue sections, and the color development reaction was stopped with purified water after 5 min of immersion. Next, sections were counterstained with hematoxylin and dehydrated with graded alcohol (75%, 95%, 100%). Sections were removed from graded alcohol to air dry and sealed with neutral resin. Immunohistochemical was observed with a microscope (×200 objectives). Three sections were selected for each group, and 10 random fields of view were selected from each section (18). Finally, statistical analysis and graphing were performed, according to the scoring results.

### AQP1, P53 and P21 protein expression

To observe the expression of AQP1, P53 and P21 protein concentrations in plasma and kidney tissue of rats in the different experimental groups. Kidney tissues and serum samples were taken from -80s°C and thawed slowly on ice. 100mg of rat kidney tissue was accurately weighed, protease inhibitor was added, and the supernatant was thoroughly ground on ice according to the operation manual. Rat serum was centrifuged at 400g for 30 seconds and assayed in strict accordance with the operation manual. The concentration of AQP1, P53, P21 and four inflammatory factors (IL-1β, IL-6, TNF-α and pNF-κB) in serum and kidney tissues were determined by rat ELISA kits, respectively.

### Real time polymerase chain reaction

Gene expression of rat kidney tissues and HK-2 cells were determined by real time PCR. Briefly, total RNA was isolated from rat kidney tissues and HK-2 cells using RNAiso Plus reagent according to the manufacturer’s protocol. RNA was reverse-transcribed (RT) to cDNA by using a Script cDNA Synthesis Kit. Quantitative RT-PCR was performed using the Bio-Rad CFX96 Optics Real-Time PCR System (Bio-Rad Laboratories, Inc., CA, USA). The following PCR conditions were used for purpose gene: 40 cycles of denaturation at 95°C for 5 s, annealing at 60°C for 30 s, and extension at 72°C for 15 s. The primer sequence was listed in **Tables 1**, **2**.

**Table 1:** Gene primer sequences of Wistar male rats.

Gene	Forward primer(5’-3’)	Reverse primer(5’-3’)
AQP1	ACCTGCTGGCCATTGACTAC	CCAGGGCACTCCCAATGAAT
P53	CGAGATGTTCCGAGAGCTGAATG	GTCTTCGGGTAGCTGGAGTG
P21	CGGGCAGTCCCTTCTAGTTCC	AATGCTTGAGCACACACGAG
NGAL	CCGACACTGACTACGACCAG	CATTGGTCGGTGGGAACAGA
KIM-1	GCCTGGAATAATCACACTGTAAG	GCAACGGACATGCCAACATAG
BAX	GTGAGCGGCTGCTTGTCT	GGTCCCGAAGTAGGAGAGGA
BCL-2	GGCATCTGCACACCTGGAT	GAGACAGCCAGGAGAAATCAAAC
Caspase-3	GGTATTGAGACAGACAGTGG	CATGGGATCTGTTTCTTTGC
FN	ATCTCCTCCCATCCACTCAA	AAACAGCCAGGCTTGCTCTGA
α-SMA	CGGGCATCCACGAAACCACC	TTCATGGTGCTGGGAGCGAGGG
TGF-β	GTGGCTGAACCAAGGAGACG	CGTGGAGTACATTATCTTTGCTGTC
GAPDH	AAGGGCTCATGACCACAGTC	GGATGCAGGGATGATGTTCT

**Table 2:** Gene primer sequence of HK-2 cell model.

Gene	Forward primer(5’-3’)	Reverse primer(5’-3’)
AQP1	CTGGGCATCGAGATCATCGG	ATCCCACAGCCAGTGTAGTCA
P53	CCGCAGTCAGATCCTAGCG	AATCATCCATTGCTTGGGACG
P21	GTGGACCTGTCACTGTCTT	GCGTTTGGAGTGGTAGAAATCTG
NGAL	TCACCTCCGTCCTGTTTAG	CTCCTTGGTTCTCCCGTA
BAX	TTCTGACGGCAACTTCAACTGG	AGGAAGTCCAATGTCCAGCC
BCL-2	TGTGGTATGAAGCCAGACCTCC	CAGGATAGCAGCACAGGATTGG
Caspase-3	GTTCATCCAGTCGCTTTGTGC	AAATTCTGTTGCCACCTTTCG
FN	GAGCTGCACATGTCTTGGGAAC	GGAGCAAATGGCACCGAGATA
α-SMA	GTGTTGCCCCTGAAGAGCAT	GCTGGGACATTGAAAGTCTCA
TGF-β	CAACAATTCCTGGCGATACCT	CAACCACTGCCGCACAAC
IL-6	GGTA CATCCTCGACGGCATCT	GTGCCTCTTTGCTGCTTTCAC
IL-1β	AACCTCTTCGAGGCACAAGG	GGCGAGCTCAGGTACTTCTG
TNF-α	CACCACTTCGAAACCTGGGA	TGTAGGCCCCAGTGAGTTCT
GAPDH	CACCCACTCCTCCACCTTTG	CCACCACCCTGTTGCTGTAG

### Cell culture and transfection

HK-2 cells (a proximal tubule epithelial cell line from a healthy human kidney), were purchased from the ATCC (Manassas, VA, USA) and incubated at 37˚C for 3 d in a humidified atmosphere containing 5% CO_2_ with DMEM-F12 medium containing 10% fetal bovine serum and 1% penicillin−streptomycin, and passaged twice per week. To minimize age-dependent variation, cells from passages 10-15 were used.

Transfection was performed when the cells reached 50% confluence. HK-2 cells were transfected with 100 nM small interfering-negative control RNA (si-NC) or si-AQP1 for 24 h using RiboFECT™CP Transfection Kit according to the manufacturer’s protocols. Following transfection, the medium was replaced with fresh medium, and after 4h, the cells were harvested for detection of transfection efficiency using RT-qPCR.

The pcDNA3.1 plasmid was purchased from Thermo Fisher Scientific and was commissioned to help construct the pcDNA3.1-AQP1 expression vector. The coding sequence of AQP1 was synthesized with reference to NCBI gene accession number: NM_198098.4, and the selected cleavage sites were NheIand XhoI; 2×10^4^ cells were plated on six-well plates and cultured for 24h; cells at 50–60% confluence were transfected with transfection reagent jetPRIME in accordance with the manufacturer’s recommendations, incubated at room temperature for 4 h and then replaced with medium. The transfected cells were harvested and verified by Western blotting and RT-qPCR.

### Cell viability assays

HK-2 cells were seeded in 96-well plates at a density of 2×10^4^ and cultured for 12 h, the cell supernatants were replaced with basal medium 1640 without serum and antibiotics and cultured for 30min after 8 h of stimulation with different concentrations of LPS. Then according to the operation instructions, a Cell Counting Kit-8 reagents were separately added to well for full reaction at 37°C for 1 h. The cell viability rates were assessed by measuring the optical density at 450 nm using a microplate reader (Thermo Fisher Scientific, Inc.).

HK-2 cells were re-planted in 96-well plates and cultured for 12 h, then cells were stimulated with LPS (12μg/ml) at different periods (1 h, 2 h, 4 h, 6 h, 8 h, 10 h, 12 h, 18 h, 24 h). Cell viability rates were assessed by measuring the optical density at 450 nm using a microplate reader. The time of LPS stimulation was determined after statistical analysis.

### Flow cytometry

Flow cytometry was performed to quantify apoptotic cells using an Annexin V-FITC/PI Apoptosis Detection kit. HK-2 cells were harvested and washed in ice-cold PBS twice, making cell suspension, taking 100μl of cell suspension into 5ml culture tube, Annexin V-FITC and PI 5μl were added to cell suspension, respectively. The double-stained with Annexin V-FITC and PI at room temperature for 15 min in the dark, 400μl of 1XBinding buffer was added and analyzed by flow cytometry within 1 h. All samples were quantitatively analyzed using a FACSC alibur flow cytometer at 488 nm emission and 570 nm excitation (BD Biosciences, San Jose, CA, USA) and analyzed by Cell Quest software (version 3.0; BD Biosciences).

### Western blot analysis

Following treatment, HK-2 cells were washed twice in cold PBS and lysed in radioimmunoprecipitation assay lysis buffer and PMSF with protease inhibitor cocktail and phosphatase inhibitors for 30 min to extract proteins. The extracted protein was crushed by ultrasonic wave, centrifuged at 14000r/min at 4 °C for 5min, and the supernatant was absorbed for the next step. The protein concentration was quantified using a BCA Protein Assay kit and samples (30 µg/lane) were separated by 12% SDS-PAGE. Blots were transferred to polyvinylidene fluoride membranes bloc, which were subsequently blocked in 5% skimmed milk diluted with TBST at room temperature for 1h and incubated overnight at 4˚C with the following primary antibodies: Anti-AQP-1 antibody (1:1000), Anti-P53 antibody (1:1000), Anti-P21 antibody (1:1500), Anti-β-actin antibody (1:2000). The PVDF membrane was washed three times with TBST at room temperature for 10 min each. Membranes were subsequently incubated with a goat anti-Rabbit IgG (1:10000) or a goat anti-mouse IgG (1:10000) conjugated to horseradish peroxidase for 1 h at room temperature. Proteins were visualized using enhanced chemiluminescence-plus reagents. The density of the bands was measured using the Image J software (version 1.45s; National Institutes of Health, Bethesda, MA, USA) and values were normalized to the densitometric values of β-actin.

### Statistical analysis

Statistical analyses were carried out using GraphPad Prism 8.0.1 software (La Jolla, CA, USA). Results were expressed as mean (±) standard deviations (S.D.). Comparisons between multiple groups were performed using one way analysis of variance (ANOVA). The two-tailed Student’s t test was used for the comparison of two groups. The statistical significance was set at *P <0.05, **P <0.01, or ***P <0.001, ****P <0.0001, or ns P >0.05.

## Results

### The transcriptional sequencing analysis of CLP-induced AKI mice and septic AKI patients

**Figure 1:**
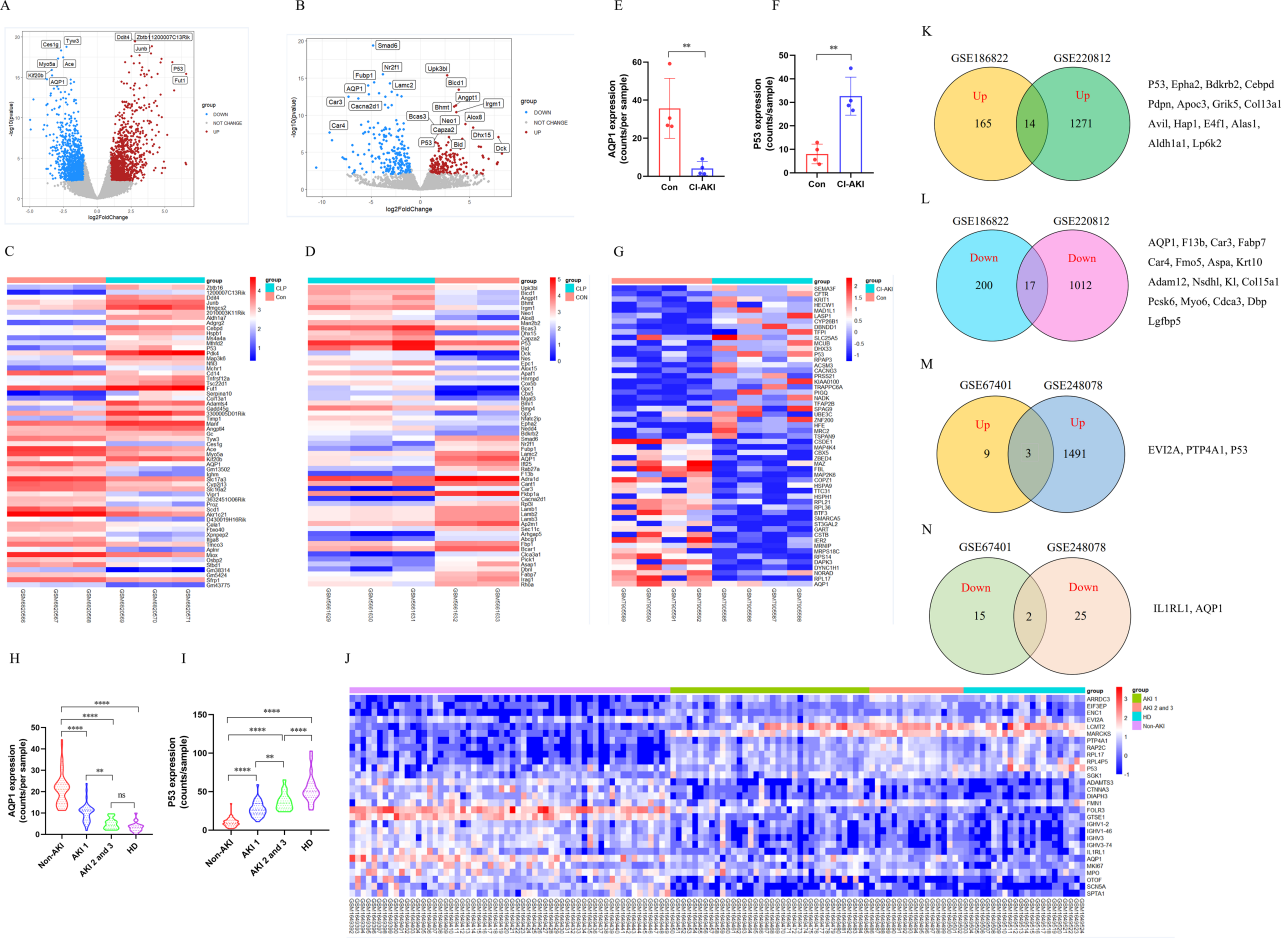
Identification and bioinformatic analysis of differentially expressed genes from GSE220812, GSE186822, GSE248078 and GSE248078. **(A)** Volcano plot of GSE220812. **(B)** Volcano plot of GSE186822. **(C)** Heatmap of GSE220812. **(D)** Heatmap of GSE186822. **(E, F)** Expression of AQP1 and P53 in renal tissues of patients with CI-induced AKI. **(G)** Heatmap of GSE248078. **(H, I)** Peripheral blood level of AQP1and P53 with systemic inflammatory response syndrome that had AKI or HD. **(J)** Heatmap of GSE67401. **(K, L)** Venn diagram of differentially expressed genes between GSE186822 and GSE220812. Up, up-regulated; Down, down-regulated. **(M, N)** Venn diagram of differentially expressed genes between GSE67401 and GSE248078. *P <0.05, **P <0.01, ***P <0.001, ****P <0.0001, or ns p >0.05.

To explore whether AQP1 and P53 are involved in AKI pathology, we performed transcriptomic analysis of renal tissue from CLP-induced AKI mice. There were 2359 DEGs after CLP-AKI in GSE220812 (**Figure 1A**). Of the DEGs (*p <*0.05 and log2FoldChange >= or <=1), there were a total of 1322 genes upregulated and 1037 genes downregulated in AKI upon CLP treatment (**Figure 1A**). Among the most significant of the genetic alterations, AQP1 was downregulated and P53 expression was upregulated in renal tissues at 24 h of CLP-AKI mice (**Figure 1C**). Homoplastically, there were 403 significantly changed genes, of which 180 were up-regulated and another 223 were down-regulated at 6 h after CLP-AKI in GSE186822 (**Figure 1B**). Meanwhile, AQP1 was downregulated and P53 was upregulated in CLP-AKI mice (**Figure 1D**). Among these significantly changed genes, 14 up-regulated and 17 downregulated genes were in common between GSE186822 and GSE220812 (**Figures 1K, L**). Most importantly, both AQP1 and P53 are included these significantly changed genes.

Next, to better understand whether AQP1 and P53 regulate the septic AKI process, we performed transcriptomic analysis of renal tissue or whole blood samples from AKI patients. As shown in (**Figures 1E, F**), renal tissues of contrast (CI)-AKI patients showed significantly lower expression of AQP1 and higher expression of P53 compared with the control group. Among the upregulated and downregulated transcripts induced by CI-AKI were AQP1 and P53 which have been shown to be associated with AKI (**Figure 1G**). Moreover, blood transcriptome analysis of systemic inflammatory response syndrome with AKI or Hemodialysis (HD) patients showed that AQP1 was dramatically decreased, whereas P53 expression was markedly elevated (**Figures 1H, I**). Meanwhile, as shown in (**Figure 1J**), AQP1 was downregulated and P53 was upregulated in systemic inflammatory response syndrome with AKI or HD patients. Among these significantly changed genes, 3 up-regulated and 2 downregulated genes were in common between GSE67401 and GSE248078 (**Figures 1M, N**). Both AQP1 and P53 are included these significantly changed genes. Surprisingly, as shown in (**Supplementary Figures S3A, B**), analysis of mRNA-seq sequencing of whole blood samples from 10 adults revealed that AQP1 expression was dramatically lower and P53 expression was markedly higher in septic AKI patients compared to controls. Indeed, among the upregulated and downregulated transcripts induced by septic AKI patients, AQP1 expression was downregulated and P53 expression was upregulated (**Supplementary Figure S3C**).

Therefore, these results suggested that AQP1 and P53 play an important role in the pathophysiologic process of sepsis induced AKI. Next, we went to explore the regulatory role of AQP1 and P53 in septic AKI by *in vivo* and *in vitro* experiments.

### Changes in renal function and histomorphology in rats at different stages of LPS-induced AKI and CLP-induced AKI

**Figure 2:**
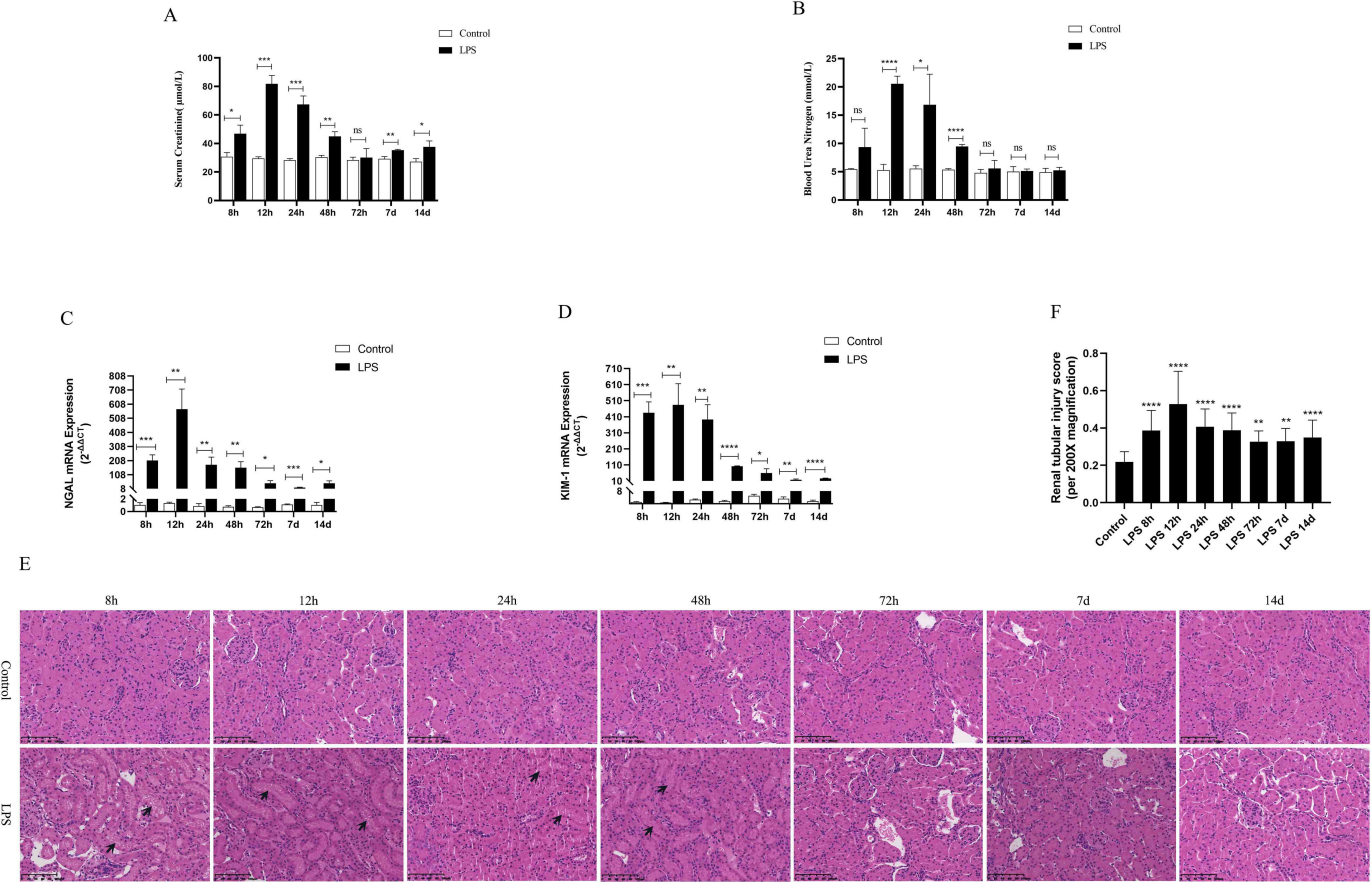
Renal function was assessed for septic AKI at various times. **(A, B)** Serum creatinine and blood urea nitrogen levels at various times in LPS-induced AKI. **(C, D)** The expression of NGAL mRNA and Kim-1 mRNA in kidney of rats with septic AKI at different periods. **(E)**The kidney tissues of rats were prepared by HE staining to observe the renal injury and morphologic analysis at various periods after LPS treatment (200×). **(F)** Quantitative graph of the tubular damage score. *P <0.05, **P <0.01, ***P <0.001, ****P <0.0001, or ns p >0.05.

Serum creatinine (SCr) and blood urea nitrogen (BUN) were utilized as criteria for the diagnosis of AKI. NGAL and KIM-1 are often used as indicators of early renal injury. On the one hand, our results *in vivo* experiments showed that SCr and BUN were 1.5 times higher than those of the control group at 8h after LPS treatment, indicating that the model of LPS-induced AKI was successfully constructed (**Figures 2A, B**). As shown in (**Figures 2A–D**), SCr and BUN levels in serum as well as NGAL mRNA and KIM-1 mRNA expression in kidney tissues were increased significantly first (8 h, 12 h) and then decreased gradually (24 h, 48 h, 72 h, 7 d, 14 d) in different times after LPS intraperitoneal injection compared with the control group. Besides, HE staining of kidney tissue sections was performed to assess the histomorphology in order to specifically observe the extent of renal injury in septic rats. As shown from (**Figures 2E, F**), compared with the control group, the basic structure of the kidney tissues was destroyed, the lumen of the renal tubules was enlarged, the epithelial cells of the renal tubules were swollen and the brush border was destroyed at (8 h, 12 h) after LPS treatment. However, there was less damage to renal tubular epithelial cells and less enlarge to renal tubules lumen in renal tissue at (24 h, 48 h, 72 h, 7 d, 14 d) after LPS treatment compared with the control group (**Figures 2E, F**). Therefore, these results demonstrated that the kidney of rats was severely damaged at 12 h after LPS treatment, while the renal tissue damage was alleviated and renal function gradually recovered at 24 h after LPS treatment (**Figures 2E, F**).

On the other hand, based on the transcriptome sequencing analysis of the CLP-induced sepsis mouse model, we will also verify the expression of AQP1 and P53 in the kidney of CLP-induced AKI rats. As shown in (**Supplementary Figures S1A–C**), SCr, BUN levels in serum as well as mRNA expression of NGAL and KIM-1significantly in renal tissues of rats were elevated at 24 h of CLP treatment compared to the sham group. These results suggested that the CLP-induced AKI model was successfully constructed. In the meantime, HE staining of kidney tissue sections reveled that there was disorganization of renal tubular structure, enlargement of tubular lumen as well as swelling and destruction of epithelial cells in CLP-induced AKI (**Supplementary Figures S1D, E**).

### Inflammation, apoptosis and interstitial fibrosis of renal were up-regulated in rats with septic AKI

**Figure 3:**
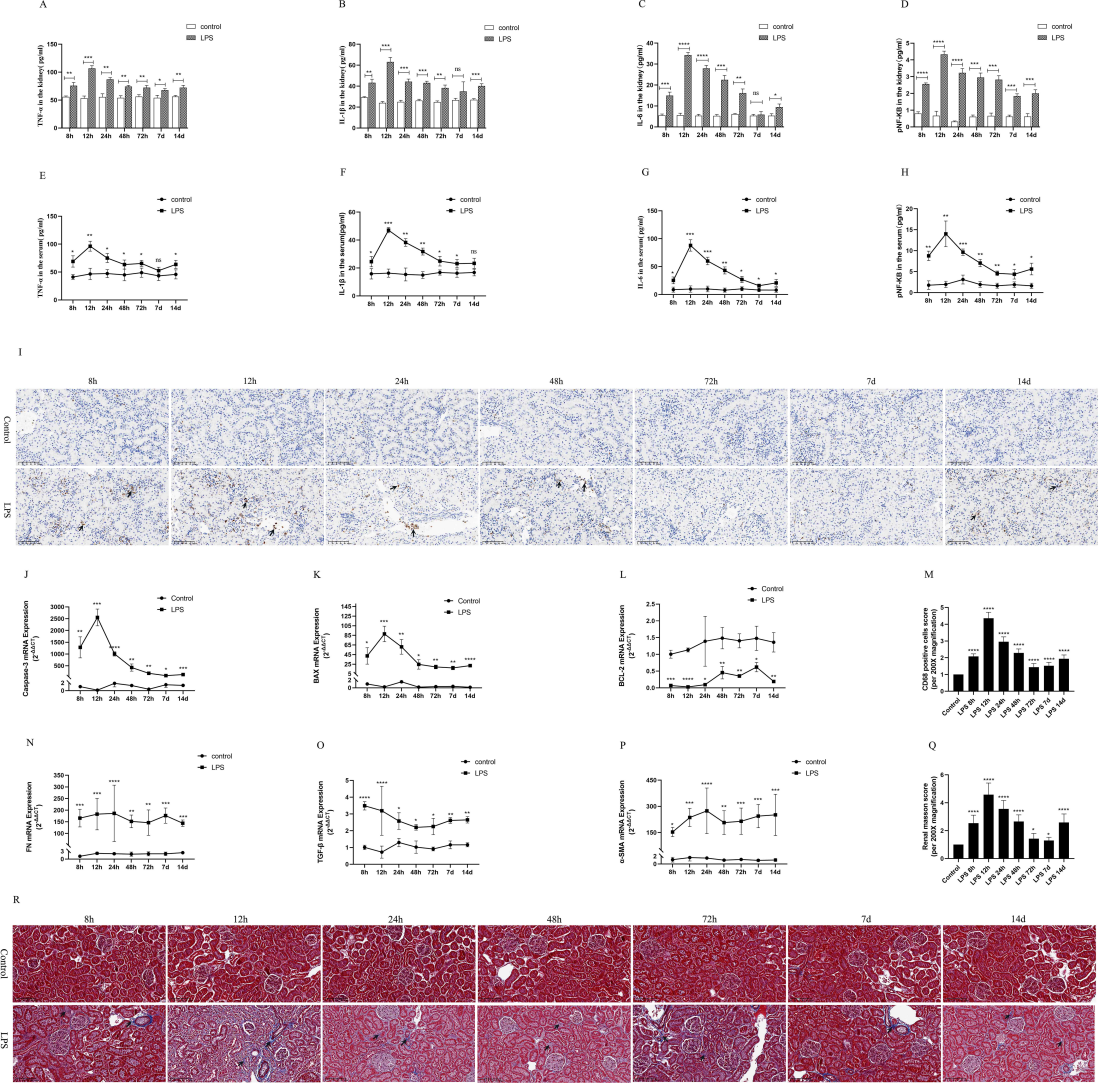
Inflammatory response, apoptosis and interstitial fibrosis during different periods of septic AKI. **(A–D)** The levels of TNF-α, IL-1β, IL-6 and pNF-κB in kidney from rats at different times after intraperitoneal injection of LPS. **(E–H)** The levels of TNF-α, IL-1β, IL-6 and pNF-κB in serum of rats during different periods of LPS-induced AKI. **(I)** The kidney tissues of rats were prepared by immunohistochemistry to observe the Number of inflammatory cell infiltration in renal tissue at various periods after LPS treatment. **(J–L)** Expression of Caspase-3 mRNA, BAX mRNA and BCL-2 mRNA of in renal tissue were assessed by RT-qPCR during different periods of septic AKI. **(M)** CD68 positive cells score was evaluated from immunohistochemistry staining. **(N–P)** Level of FN mRNA, TGF-β mRNA and α-SMA mRNA of in renal tissue were evaluated by RT-qPCR at different periods in LPS induced AKI. **(Q)** Renal interstitial fibrosis was evaluated from Masson staining. **(R)** The kidney tissues from rats were prepared by Masson staining at different times after LPS treatment for analysis of the process of renal fibrosis (200×). Blue areas indicate the abundance of fibrosis of renal tissue and red areas display the absence of fibrosis. *P <0.05, **P <0.01, ***P <0.001, ****P <0.0001, or ns p >0.05.

To observe the expression of inflammatory response in the LPS group, we used ELISA kits to detect the levels of inflammatory cytokines in kidney tissues and serum of rats, mainly including IL-6, IL-1β, TNF-α and pNF-κB. The results showed that the levels of renal tissues and serum inflammatory factors (IL-6, IL-1β, TNF-α and pNF-κB) peaked at 12 h after LPS treatment and decreased at (24 h, 48 h, 72 h, 7 d, 14 d) after LPS treatment (**Figures 3A–H**). In addition, CD68 is frequently used as a macrophage (inflammatory cell) marker. Immunohistochemical results showed that renal tissue was infiltrated with a large number of inflammatory cells at 12 h after LPS treatment and diminished at (24 h, 48 h, 72 h, 7 d, 14 d) compared to the control group (**Figures 3I, M**).

Caspase-3 and BAX were regard as indicators of renal apoptosis. BCL-2, by contrast, is one of the anti-apoptotic markers. Our experimental results showed that renal Caspase-3 and BAX mRNA expression was significantly increased at 8h after LPS treatment and reached its peak at 12h compared with the control group (**Figures 3J, K**). In the following times (24 h, 48 h, 72 h, 7 d, 14 d), Caspase-3 and BAX mRNA expression decreased, but remained higher than that of the control group and was statistically significant (**Figures 3J, K**). Meanwhile, LPS treatment significantly diminished the expression of BCL-2 mRNA in kidney tissues of rat compared with the control group (**Figure 3L**). These results indicated that LPS via increased apoptosis genes and inhibition of antiapoptotic genes expression at different times in sepsis AKI, eventually caused kidney tissues apoptosis continues.

FN, α-SMA and TGF-β were used as classical indexes of renal interstitial fibrosis. Renal fibrosis factors from LPS group continuously increased compared with the control group (**Figures 3N–P**). Masson staining of rat kidney tissues also showed that renal interstitial fibrosis increased significantly at different periods (8 h, 12 h, 24 h, 48 h, 72 h, 7 d, 14 d) of septic AKI (**Figures 3Q, R**). These results demonstrated that renal interstitial fibrosis is not a time-dependent reduction in LPS-induced AKI.

Moreover, the results *in vivo* experiments showed that the inflammatory response was enhanced in rats during CLP-induced AKI, which was mainly manifested by greatly increased levels of inflammatory factors (IL-1β, pNF-kB, TNF-α, IL-6) in renal tissues and serum (**Supplementary Figures S2D, E**). Similarly, rat kidney tissue was infiltrated with a large number of inflammatory cells as well as increased levels of renal interstitial fibrosis were assessed by Photomicrographs at 24 h of CLP treatment (**Supplementary Figures S2F–I**).

### Changes in AQP1 protein and mRNA levels in rats at various points of septic AKI

**Figure 4:**
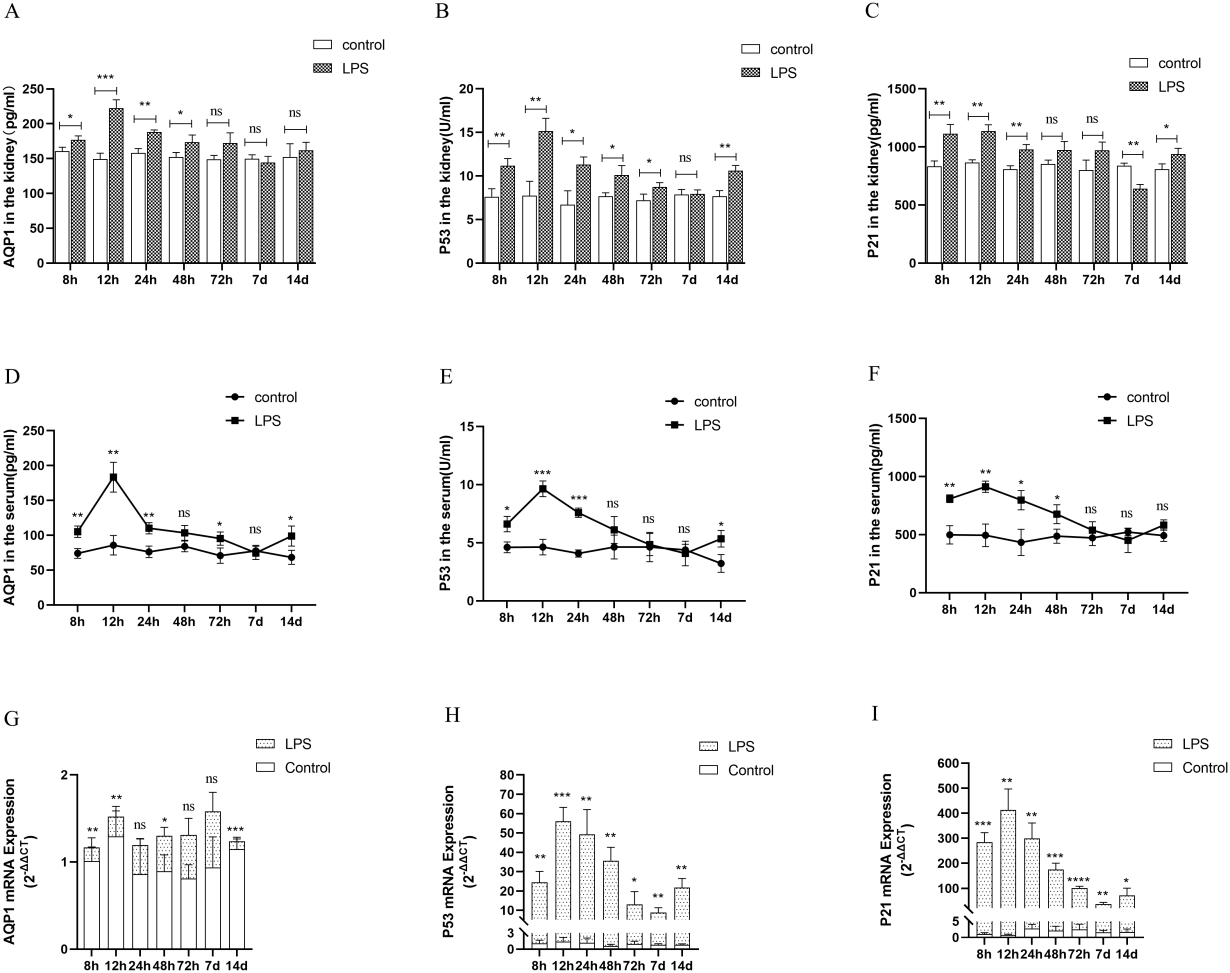
Expression of AQP1, P53 and P21 proteins and mRNAs at different times of septic AKI. **(A–F)** Expression of AQP1, P53 and P21 proteins in kidney tissues and serum from different groups after LPS treatment. **(G–I)** Expression of AQP1, P53 and P21 mRNA in renal tissues from different times of septic AKI. *P <0.05, **P <0.01, ***P <0.001, ****P <0.0001, or ns p >0.05.

In the animal experiments, we established an AKI model by intraperitoneal injection of LPS and found that AQP1 protein expression was increased in kidney tissues and serum of rats at 8 h after LPS treatment and peaked at 12 h, then decreased at 24 h and reached the lowest level at 7 d (**Figures 4A, D**). Similarly, the protein levels of P53 and P21 in kidney tissues and serum of rats were increased at 12 h and decreased at (24 h, 48 h, 72 h and 7 d) in LPS-induced AKI (**Figures 4B, C, E, F**). In addition, the mRNA levels of AQP1, P53 and P21 in kidney tissues of rats were detected by RT-qPCR. AQP1 mRNA expression in kidney tissues in rats was continuously decreased at different periods in LPS induced AKI compared with the control group (**Figure 4G**). However, P53 mRNA and P21 mRNA expression were firstly increased and then decreased, which was consistent with P53 and P21 protein expression changes (**Figures 4H, I**).

Homoplastically, the expression of AQP1, P53 and P21 protein were all significantly elevated in renal tissues and serum of the CLP group compared with the sham group (**Supplementary Figures S2A, B**). The gene expression of P53 and P21 was consistent with the trend of protein expression, while AQP1 mRNA expression was reduced in rat kidney tissues at 24 h of CLP treatment (**Supplementary Figure S2C**). This result suggested that the elevated expression of AQP1 protein changes in renal tissues during septic AKI is not dependent on the upregulation of its gene, and most likely exerts a renoprotective effect as a result of stress elevation, while P53 and P21 protein changes may be caused by gene changes.

In conclusion, the combination of all the above results indicated that the most significant changes in renal function, renal inflammation, apoptosis and fibrosis levels as well as protein and gene expression of AQP1, P53, and P21 were observed in rats at 12h of LPS-induced AKI. Therefore, we will choose the septic AKI model treated with LPS for 12h to go for the subsequent experiments.

### Silencing of AQP1 promotes renal inflammation, apoptosis and interstitial fibrosis in septic AKI rats through up-regulation of P53 expression

**Figure 5:**
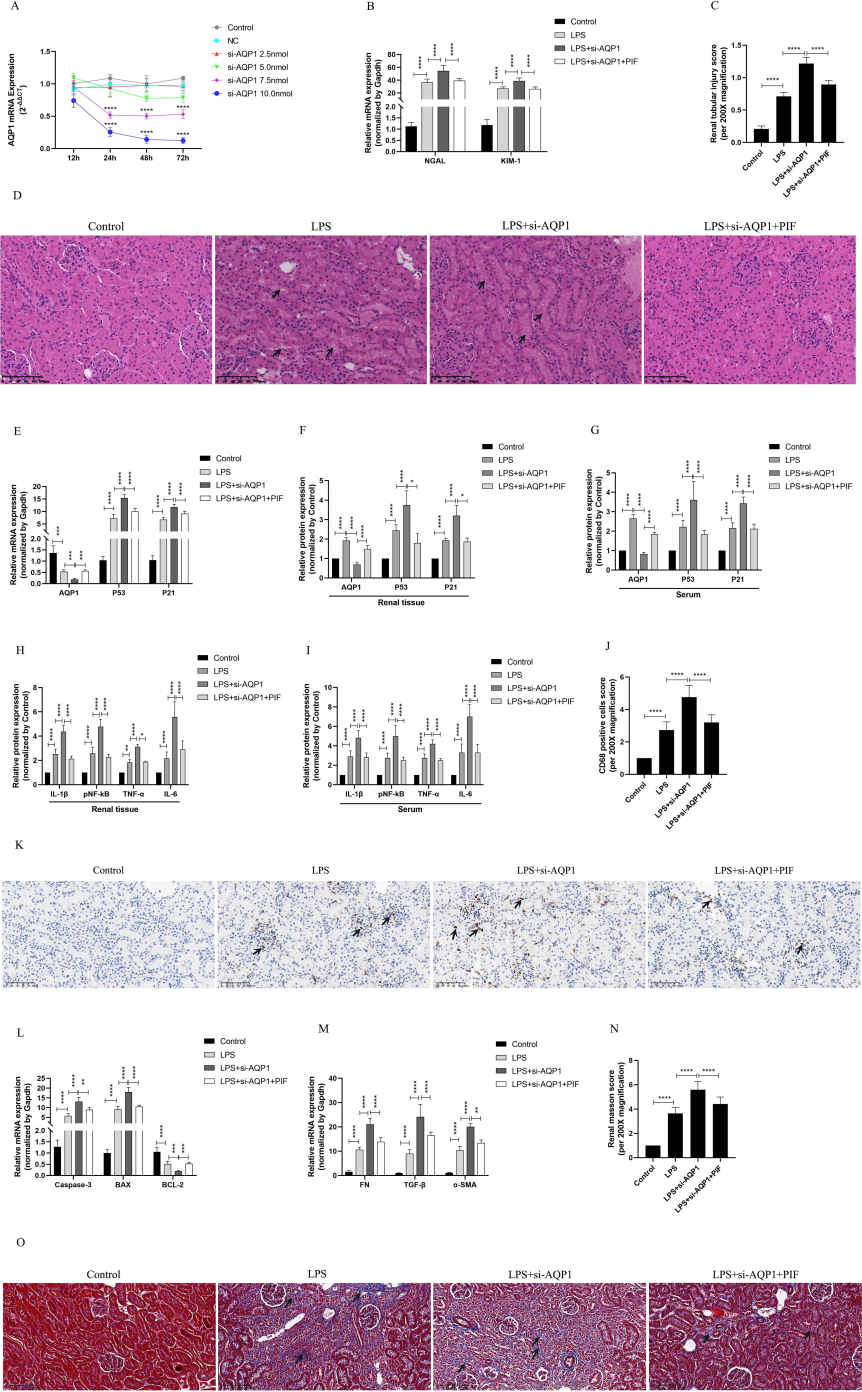
Validation of silencing AQP1 in septic AKI rats exacerbates renal injury by upregulating P53. **(A)** Renal AQP1 silencing in rats caused by tail vein injection different concentrations of si-AQP1. **(B)** Expression of NGAL mRNA and KIM-1 mRNA in kidney at various group in septic AKI. **(C)** Quantitative graph of the tubular damage score. **(D)** Injury pathology of the renal tissue of rats with LPS-induced AKI. Photomicrographs of HE stained kidney sections (200×). **(E)** Expression of AQP1 mRNA, P53 mRNA and P21 mRNA in kidney were determined by RT-qPCR. **(F, G)** Expression of AQP1, P53 and P21 proteins in kidney and serum of rats. **(H, I)** Expression of inflammatory mediators in kidney and serum of rats, mainly includingIL-1β, pNF-kB, TNF-α and IL-6. **(J)** CD68 positive cells score was evaluated from immunohistochemistry staining at various group. **(K)** The kidney tissues of rats were prepared by immunohistochemistry to observe the abundance of inflammatory cell infiltration in renal tissue in LPS-induced AKI. **(L, M)** Kidney tissue apoptosis and fibrosis gene expression evaluated by RT-qPCR in septic AKI rats. **(N)** Renal interstitial fibrosis was evaluated at various group in LPS-induced AKI. **(O)** The kidney tissues from rats were prepared by Masson staining at various group in LPS-induced AKI (200×). *P <0.05, **P <0.01, ***P <0.001, ****P <0.0001, or ns=not significant.

We used various concentrations of si-AQP1 to silence rat kidney AQP1 by tail vein injection. The results showed that 10 nmol of si-AQP1 had the most significant inhibitory effect in 72 h (**Figure 5A**). RT-qPCR results showed that the mRNA levels of NGAL and KIM-1 were higher in the LPS+si-AQP1 group compared with the LPS group, and inhibition of P53 via Pifithrin-α (PIF, a P53 inhibitor) reversed this phenomenon (**Figure 5B**). Meanwhile, HE staining showed that inhibition of P53 by PIF significantly attenuated silencing AQP1-mediated renal tubular structural disorganization and tubular epithelial cell swelling (**Figures 5C, D**). These results demonstrated that silencing AQP1 further aggravates LPS-induced renal injury, whereas inhibiting P53 expression partially alleviates renal injury, indirectly suggesting that AQP1 plays a renoprotective role in septic AKI.

Our results *in vivo* experiments showed further elevation of P53 and P21 proteins and genes in kidney tissues and serum of septic AKI rats after silencing of AQP1, which was reversed by inhibition of P53 (**Figures 5E–G**). This result suggested that silencing AQP1 participates in the pathologic injury process of septic AKI by promoting the upregulation of P53 expression. In addition, the levels of inflammatory factors, mainly including IL-1β, pNF-κB, TNF-α and IL-6, were significantly higher in serum and renal tissues of rats in the LPS+si-AQP1 group compared with those in the LPS group (**Figures 5H, I**). At the same time, immunohistochemical results also showed that inflammatory cell infiltration in renal tissue was more abundant in the LPS+si-AQP1 group compared with the LPS group (**Figures 5J, K**). Moreover, RT-qPCR results showed that the expression of pro-fibrotic and pro-apoptotic genes was markedly elevated, while the expression of anti-apoptotic genes was diminished in renal tissues of the LPS+si-AQP1 group compared with the LPS group (**Figures 5L, M**). Masson staining showed that silencing AQP1 aggravated the degree of interstitial fibrosis in renal tissue of septic AKI rats (**Figures 5N, O**). However, inhibition of P53 by PIF on the basis of LPS+si-AQP1 group significantly alleviated renal inflammatory cell infiltration, apoptosis level and interstitial fibrosis in septic AKI rats (**Figures 5E–O**). Thus, these results demonstrated that silencing AQP1 promotes renal inflammation, apoptosis and interstitial fibrosis in septic AKI rats by upregulating P53 expression.

### Silencing AQP1 aggravates cellular injury by up-regulating P53 expression in LPS-induced HK-2 cells

**Figure 6:**
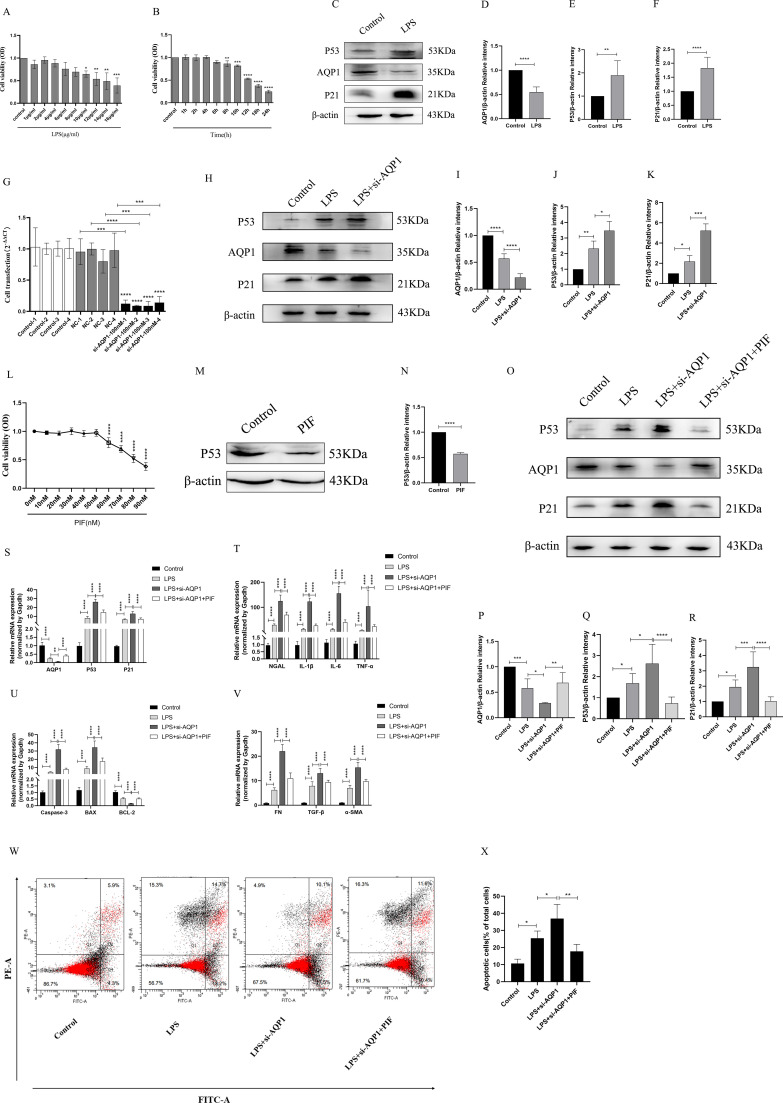
Silencing AQP1 exacerbates LPS-induced HK-2 cell injury by upregulating P53 expression. **(A, B)** HK-2 cells viability was evaluated by CCK8. **(C–F)** Expression of AQP1, P53 and P21 protein was detected by western blot in LPS stimulated HK-2 cells. **(G)** 100nM of si-AQP1 was used to silence AQP1 gene. **(H–K)** P53 and P21 protein expression was significantly elevated after silencing AQP1. **(L)** Effects of different concentrations of PIF (P53 inhibitor) on HK-2 cell viability. **(M, N)** PIF significantly inhibited P53 protein expression. **(O–R)** Expression of AQP1, P53 and P21 protein was detected by western blot at various group in LPS stimulated HK-2 cells. **(S)** Level of AQP1, P53 and P21 gene was detected at various groups in LPS stimulated HK-2 cells. **(T–V)** Level of NGAL, IL-1β, IL-6, TNF-α, Caspase-3, BAX, BCL-2, FN, TGF-β and α-SMA gene was detected by RT-qPCR at various group in LPS stimulated HK-2 cells. **(W, X)** Apoptosis level in LPS induced HK-2 cells was detected by flow cytometry. *P <0.05, **P <0.01, ***P <0.001, ****P <0.0001, or ns=not significant.

First of all, different concentrations of LPS (1μg/ml, 2μg/ml, 4μg/ml, 6μg/ml, 8μg/ml, 10μg/ml, 12μg/ml, 14μg/ml, 16μg/ml) were used to stimulate HK-2 cells for 8 h (**Figure 6A**). It was found that HK-2 cell viability was decreased in a gradient dependent manner (10μg/ml,12μg/ml,14μg/ml,16μg/ml) and HK-2 cells viability decreased significantly at 12μg/ml of LPS. Next, we choose 12μg/ml of LPS stimulate the HK-2 cells in different time (1 h, 2 h, 4 h, 6 h, 8 h, 10 h, 12 h, 18 h, 24 h). Our results suggested that cell viability continued to decreased in the time gradient of and reduced rapidly at 12 h (**Figure 6B**). These results confirmed that LPS induced HK-2 cell viability decreased in a concentration-dependent and time-dependent manner. Therefore, we finally determined 12ug/mL of LPS to stimulate HK-2 cells for 12 h *in vitro* experiment.

As shown in (**Figures 6C–F**), AQP1 protein expression was decreased, while P53 and P21 protein expression was elevated at 12h of LPS incubation of HK-2 cells. Subsequently, we significantly succeeded in knocking down AQP1 expression in HK-2 cells by using 100nM si-AQP1 (**Figure 6G**). Importantly, Western blot and RT-qPCR results showed that silencing AQP1 further contributed to elevated P53 and P21 protein and mRNA expression (**Figures 6H–K**). Furthermore, we found that 50nM of PIF significantly inhibited P53 protein and gene expression without affecting cell viability (**Figures 6L–N**).

In addition, the results showed that AQP1 protein and mRNA levels were elevated, while P53 and P21 protein and mRNA expression were reduced in the LPS+si-AQP1+PIF group compared with the LPS+si-AQP1 group (**Figures 6O–S**). Meanwhile, the mRNA levels of NGAL, inflammatory factors (IL-1β, TNF-α and IL-6), pro-apoptosis (Caspase-3, BAX) and pro-fibrosis (FN, TGF-β, α-SMA) were significantly higher in the LPS+si-AQP1 group compared to the LPS group, which was reversed by inhibition of P53 (**Figures 6T–V**). Similarly, Flow cytometry results also showed that silencing of AQP1 further enhanced the level of apoptosis, whereas inhibition of P53 level by PIF targeting on the basis of LPS+si-AQP1 significantly reversed LPS-induced apoptosis in HK-2 cells (**Figures 6W, X**). Thus, these results demonstrated that silencing AQP1 exacerbates the levels of cellular inflammatory factors, apoptosis and fibrosis by mediating the upregulation of P53 expression in LPS-induced HK-2 cells.

### Overexpression of AQP1 attenuates renal inflammation, apoptosis and interstitial fibrosis in septic AKI rats through inhibiting P53 expression

**Figure 7:**
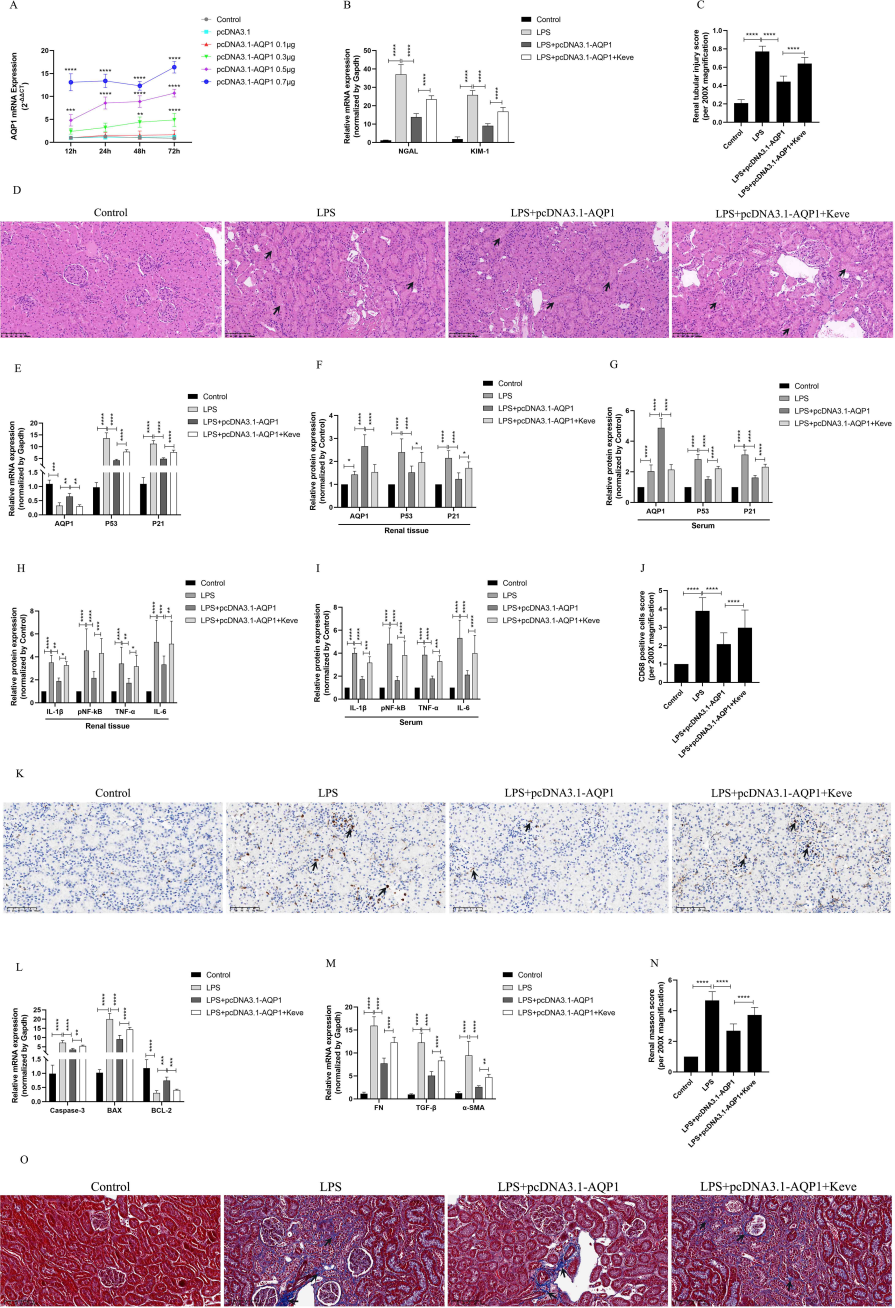
Overexpression of AQP1 was verified in septic AKI rats to alleviate renal injury by inhibiting P53. **(A)** Different concentrations of pcDNA3.1-AQP1 injected via tail vein lead to AQP1 overexpression in rat kidney. **(B)** Expression of NGAL mRNA and KIM-1 mRNA in kidney at various group in septic AKI. **(C)** Quantitative graph of the tubular damage score. **(D)** Injury pathology of the renal tissue of rats with LPS-induced AKI. Photomicrographs of HE stained kidney sections (200×). **(E)** Expression of AQP1 mRNA, P53 mRNA and P21 mRNA in kidney were determined by RT-qPCR. **(F, G)** Expression of AQP1, P53 and P21 proteins in kidney and serum of rats at various groups in Endotoxin induced AKI. **(H, I)** Expression of IL-1β, pNF-kB, TNF-α and IL-6 in kidney and serum of rats. **(J)** CD68 positive cells score was evaluated from immunohistochemistry staining at various group. **(K)** The kidney tissues of rats were prepared by immunohistochemistry to observe the Number of inflammatory cell infiltration in renal tissue in LPS-induced AKI. **(L, M)** Expression of Caspase-3 mRNA, BAX mRNA, BCL-2 mRNA, FN mRNA, TGF-βmRNA and α-SMA mRNA in renal tissue at various groups in septic AKI **(N)** Renal interstitial fibrosis was evaluated at various group in LPS-induced AKI. **(O)** The kidney tissues from rats were prepared by Masson staining at various group in LPS-induced AKI (200×). *P <0.05, **P <0.01, ***P <0.001, ****P <0.0001, or ns=not significant.

*In vivo* experimental results showed that 0.7μg of pcDNA3.1-AQP1 was more effective in overexpression of AQP1 in rat kidney at 72 h (**Figure 7A**). RT-qPCR results showed that overexpression of AQP1 significantly attenuated the levels of mRNA of NGAL and KIM-1 in renal tissues of septic AKI rats, while up-regulation of P53 expression by Kevetrin hydrochloride (Keve, a P53 agonist) restored the gene levels of NGAL and KIM-1 (**Figure 7B**). Simultaneously, HE staining followed by light microscopy showed that renal tubular structural disorganization and tubular epithelial cell swelling were attenuated in the LPS+ pcDNA3.1-AQP1 group compared with the LPS group, while up-regulation of P53 reversed this phenomenon (**Figures 7C, D**). These results suggested that overexpression of AQP1 significantly alleviates LPS-induced AKI, whereas up-regulation of P53 expression exacerbates renal injury.

Interestingly, our results showed that overexpression of AQP1 significantly suppressed the levels of P53 and P21 proteins and genes in renal tissues and serum of septic AKI rats, and up-regulation of P53 reversed this result (**Figures 7E–G**). Furthermore, overexpression of AQP1 significantly alleviated the levels of inflammatory factors in serum and renal tissues of rats as well as reduced the number of inflammatory cell infiltration in renal tissues, while up-regulation of P53 exacerbated the inflammatory response in septic AKI (**Figures 7H–K**). RT-qPCR results showed that the expression of pro-fibrotic (FN, TGF-β, α-SMA) and pro-apoptotic genes (Caspase-3, BAX) was significantly reduced and the expression of anti-apoptotic genes (BCL-2) was elevated in the kidneys of the LPS+pcDNA3.1-AQP1 group compared with the LPS group (**Figures 7L, M**). Masson staining was performed and found that overexpression of AQP1 attenuated renal fibrosis in septic AKI rats (**Figures 7N, O**). However, up-regulation of P53 on the basis of LPS+ pcDNA3.1-AQP1 significantly promoted the degree of LPS-induced renal tissue fibrosis and the level of apoptosis (**Figures 7N, O**). Accordingly, these results demonstrated that overexpression of AQP1 attenuates renal inflammatory response, apoptosis and interstitial fibrosis in septic AKI by inhibiting P53 expression.

### Overexpression of AQP1 attenuates cellular injury via inhibiting P53 expression in LPS-induced HK-2 cells

**Figure 8:**
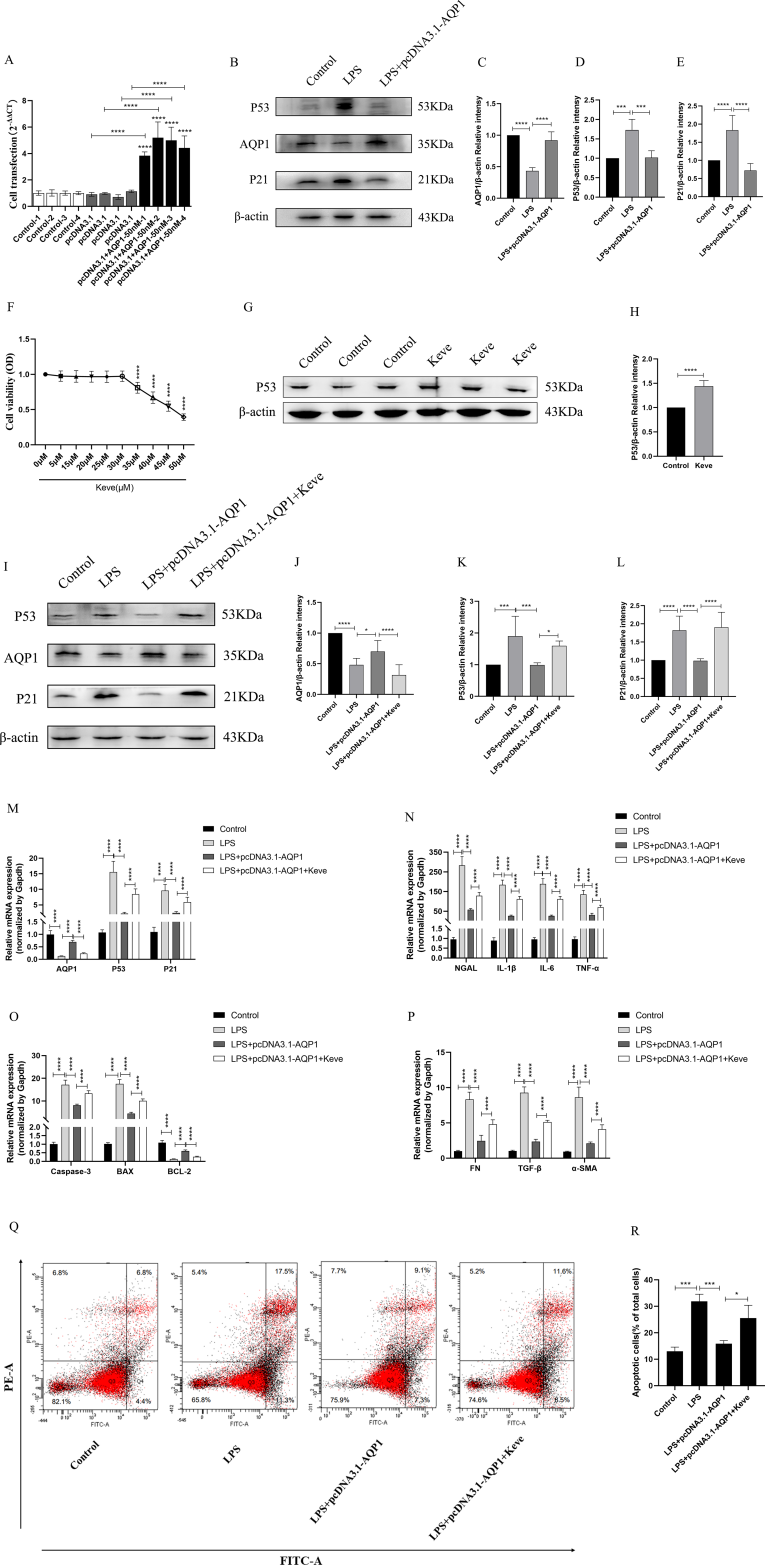
Overexpression of AQP1 attenuates LPS-induced HK-2 cell injury by inhibiting P53 expression. **(A)** 50nM of pcDNA3.1-AQP1 was used to Transfection of HK-2 cells. **(B–E)** Overexpression of AQP1 significantly reduced P53 and P21 protein expression **(F)** Effects of different concentrations of Keve (P53 agonists) on HK-2 cell viability. **(G, H)** Keve successfully upregulated P53 protein expression. **(I–L)** Expression of AQP1, P53 and P21 protein was detected by western blot at various group in LPS stimulated HK-2 cells. **(M)** Level of AQP1, P53 and P21 gene was detected at various groups in LPS stimulated HK-2 cells. **(N–P)** Level of NGAL, IL-1β, IL-6, TNF-α, Caspase-3, BAX, BCL-2, FN, TGF-β and α-SMA genes was detected by RT-qPCR at various group in LPS stimulated HK-2 cells. **(Q, R)** Apoptosis level at various groups in LPS induced HK-2 cells was detected by flow cytometry. *P <0.05, **P <0.01, ***P <0.001, ****P <0.0001, or ns=not significant.

To validate *in vitro* experiments that overexpression of AQP1 exerted a cytoprotective effect by inhibiting P53 expression in LPS-stimulated HK-2. Firstly, HK-2 cells were transfected with 50nM pcDNA3.1-AQP1 and successfully constructed AQP1 overexpression HK-2 cell line (**Figure 8A**). Western blot results showed that overexpression of AQP1 significantly inhibited the expression of P53 and P21 protein, which was consistent with the results of *in vivo* experiments (**Figures 8B–E**). Next, different concentrations of Keve were used to culture HK-2 cells and it was found that 30μM Keve did not affect cell activity and significantly upregulated P53 protein expression (**Figures 8F–H**). As shown in (**Figures 8I–M**), AQP1 protein and gene expression was reduced in the LPS+ pcDNA3.1-AQP1+Keve group compared to the LPS+ pcDNA3.1-AQP1 group, whereas P53 and P21 protein as well as gene expression were elevated. Furthermore, RT-qPCR results showed that overexpression of AQP1 significantly reduced the levels of cellular NGAL, inflammatory factors, pro-apoptotic genes and pro-fibrotic genes in LPS-induced HK-2 cells, while up-regulation of P53 levels by Keve targeting significantly reversed the cytoprotective effects of AQP1 (**Figures 8N–P**). Analogously, flow cytometry results also showed that overexpression of AQP1 significantly attenuated apoptosis levels in LPS-stimulated HK-2 cells, whereas up-regulation of P53 restored LPS-induced cell damage (**Figures 8Q, R**). Accordingly, these results confirmed that overexpression of AQP1 in LPS-induced HK-2 cells exerts a cytoprotective effect by attenuating the levels of cellular inflammatory factors, apoptotic genes and pro-fibrotic genes through inhibition of P53 expression.

## Discussion

At present, a large number of researches have elaborated the mechanism of septic AKI, mainly including inflammatory response imbalance, renal tubular epithelial necrosis and apoptosis, oxidative stress and microcirculation disorders, etc. (17). Numbers of previous *in vivo* studies on septic AKI mainly focused on the peak of inflammation and injury, namely the acute phase of septic AKI (19). Therefore, researchers mainly constructed acute stage models of septic AKI. Unfortunately, the dynamic course of post-traumatic recovery in septic AKI is poorly studied. Therefore, this study was design to explored the dynamic process of septic AKI in the acute and post-injury recovery periods. Our experimental AKI model in rats showed that 12 h of LPS treatment was the acute injury stage of septic AKI, 24 h-7 d was the recovery stage after acute injury, and 7d-14 d may be the early stage of the development of CKD after AKI. These findings may provide novel ideas for the diagnosis and treatment of septic AKI and for the development of CKD after AKI.

Inflammatory storm is one of the main mechanisms of high mortality in septic AKI (17). In this study, the dynamic process of LPS induced AKI was researched by intraperitoneal injection LPS. It was found that the levels of inflammatory factors (IL-1β, pNF-κB, TNF-α and IL-6) in renal and serum peaked at 12 h after LPS treatment and returned to baseline at (24 h, 48 h, 72 h, 7 d and 14 d). The results suggested that the peak of inflammatory response and the most serious and vulnerable period of renal injury was 12 h of LPS treatment during septic AKI. In addition, we found that the expression of P53 was also significantly increased along with the upregulation of inflammatory factors. Therefore, we hypothesized that upregulation of P53 directly or indirectly promotes inflammatory response in endotoxemia AKI. Lowe et al. (20) demonstrated by transcriptional analysis that p53 regulates pro-inflammatory gene responses in human macrophages. Mechanistically, p53 rapidly and efficiently induces IL-6 by binding to its promoter and decreases IL-6 levels in response to p53 inhibition. Zhang et al. (21) also reported in a model of inflammatory response in fibroblast-like synoviocytes that p53 mainly regulates IL-6 production. In addition, AKI-induced up-regulation of P53 may indirectly mediate inflammation by activating oxidative stress. Homsi and colleagues (22) reported that the expression of P53 in rat kidney significantly increased and activated oxidative stress after glycerin induced AKI, aggravating renal tubular injury. PIF inhibition of P53 alleviates renal injury and significantly reduces tubule injury. These researches are consistent with our *in vivo* and vitro results, namely, silencing AQP1 promotes inflammatory response in septic AKI by upregulating P53 expression. Notably, intraperitoneal injection of PIF inhibited P53 expression in rats significantly reduced the levels of inflammatory factors in LPS-induced AKI. Moreover, the *in vitro* results of LPS-stimulated HK-2 were consistent with the *in vivo* results.

Interestingly, inflammatory factors also regulate the accumulation of P53 protein. Bonda et al. (23) have demonstrated that IL-6 knockout inhibits the accumulation of p53 in mouse myocardium associated with aging. Bialuk and colleagues (24) found that IL-6 deficiency attenuated p53 protein accumulation in hippocampus of aged male mice. Summary of these findings shown that P53 and inflammatory response are mutually influential. In brief, the expression of P53 in cytoplasm and nucleus is up-regulated after tissue or cells are subjected to damaged or stimulated, which leads to a large number of inflammatory factors to be released extracellular by binding and activating inflammatory genes. Extracellular inflammatory factors will be regard as a stimulator return continue to promote P53 expression, which eventually form a P53-inflammatory factor-P53 closed-loop pathway. Therefore, inhibition of P53 will be disrupted this closed loop and reducing kidney injury. As shown in our results, silencing AQP1 exacerbated the septic AKI inflammatory response by upregulating P53. Overexpression of AQP1 decreased inflammatory factor secretion and attenuated renal inflammatory cell infiltration abundance by inhibiting P53 expression. In contrast, Sutton et al. (25) demonstrated that p53 exerts a protective effect by reducing the extent and duration of inflammation and promotes an anti-inflammatory M2 macrophage phenotype in mouse renal tissues after ischemic injury. We speculate that this may be related to the type and pathological mechanism of AKI, the species of mice and the type of target cells.

Previous studies have shown that apoptosis occurs at AKI as an outcome of irreversible injury (26). Apoptosis may be the main cause of renal injury in septic AKI (27, 28). In our study, apoptosis occurred significantly in renal tissues in sepsis induced AKI and lasted for 14 d. This result is consistent with previous findings. Therefore, we hypothesized that LPS-induced renal apoptosis in septic AKI has the following mechanisms: one is that stress molecules stimulate mitochondria and reduce ATP production, which is mainly affected by changes in intracellular chemical information (29). The other includes TNF-α, which are influenced by messages from extracellular stimuli. Investigators (30) found that apoptosis persisted for 48 h in renal tissues of LPS-induced AKI mice, whereas knockdown of TNFR1 significantly reduced apoptosis and alleviated renal injury. Our results also showed that TNF-α expression was significantly increased in renal tissue and serum of septic AKI. Therefore, LPS mediated renal tissue apoptosis through inflammatory factors, mainly TNF-α, is an important pathological pathway of renal injury in sepsis mediated AKI. Thirdly, P53 induces apoptosis. P53 mediated tubular cell death as a major contributor to the pathological mechanism of AKI has been reported (31). For instance, transcriptional activity of p53 plays an indispensable role in renal tubule apoptosis in cisplatin nephrotoxicity (32). Mechanistically, p53 activation promotes apoptosis in renal tubular epithelial cells through upregulation of apoptosis regulators and pro-apoptotic proteins in cisplatin nephrotoxicity (33, 34). Furthermore, p53 activation-mediated upregulation of BAX and SIVA1 have been shown to be a primary factor in renal tubular epithelial cell apoptosis after IR (35, 36). In addition to transcriptionally induced pro-apoptotic function, P53 also leads to significant upregulation of apoptotic factors in the cytoplasm through directly binding to BCL-2 protein, which ultimately activates a series of apoptotic cascade responses (37). Collectively, these findings support the results of our *in vivo* and *in vitro* experiments that LPS-induced downregulation of AQP1 in renal tissues or HK-2 cells concomitantly stimulated significant upregulation of P53 and apoptotic molecules (BAX mRNA, Caspase-3 mRNA). In addition, inhibition of P53 expression markedly down-regulated pro-apoptotic gene (BAX mRNA, Caspase-3 mRNA) and up-regulated anti-apoptotic gene (Bcl-2 mRNA) in PIF pretreated or co-cultured HK-2 cells.

Previous studies on renal fibrosis mainly focused on ischemic AKI, while a few studies on renal fibrosis in the acute phase of septic AKI (1, 17). Plotnikov et al. (38) observed the mechanism of kidney cell damage in newborn and adult rats after 28 days of LPS-mediated AKI model, and estimated that renal fibrosis in adult kidneys was significantly higher, while such changes in neonatal kidneys were less serious. Moreover, the tolerance of adult rat kidney cells to oxidative stress was worse than that of newborn rats, and it were more affected by long-term pathological consequences such as fibrosis. Although LPS-mediated septic AKI renal fibers were observed after 28 d in both models, lacking the early stage of renal fibrosis observed. In our study, it was found that the renal interstitial fibrosis factors (FN mRNA, α-SMA mRNA and TGF-β) were significantly increased and their expression did not decreased with time extension at different periods in septic AKI. Moreover, we demonstrated that overexpression of AQP1 exerted renoprotective effects by reducing the degree of fibrosis through inhibition of P53 expression.

The following explanations are made for renal fibrosis in different periods of septic AKI. The role of inflammation in the process of AKI transition to CKD has received a lot of attention (17). What’s more, the kidney fibrosis was involved in AKI to CKD transition and considered as the early change (17). Therefore, 1) The continuous stimulation of inflammatory cytokine storm may be the main cause of acute renal fibrosis in septic AKI. Mouse nephropathy models (39) have shown that increased IL-1 activates TLR/IL-1R on the proximal tubules and promotes interstitial fibrosis. Homoplastically, IL-1 plays an important role in the development of fibrosis using a novel human renal fibrosis model (40). Ying et al. (41) found that intracellular ROS production and activation of the stat1 pathway in proximal renal tubule cells mediated the upregulation of IL-1β and TGF-β, leading to renal interstitial fibrosis. 2) Incomplete repair of renal tissue during recovery after LPS-induced AKI contributes to uncontrolled chronic inflammatory response after AKI that promotes interstitial fibrosis. Yu and his colleagues (42) illustrated that the maladaptive response of renal tubular epithelial cells after AKI ultimately leads to renal fibrosis and dysfunction. Similarly, H. Jin and colleagues (43) demonstrated knockout of myeloid differentiation 88 (Myd88) inhibited epithelial cell-specific innate immune signaling and significantly reduced fibrosis and renal tubular injury. Taken together, these results suggested that continuous stimulation of inflammatory factors leads to renal fibrosis in AKI, while inhibition or reduction of inflammatory factor production mitigates renal fibrosis. Indeed, Clinical data from an antifibrotic drug development program also suggest that reducing NLRP3 activation attenuates fibrosis in a model of unilateral ureteral obstruction (44). 3) P53 was involved in renal incomplete repair and fibrosis after AKI. Evidence from investigators suggests that p53 activation and the assembly of SMAD3 play a crucial role in the transcription of fibrosis genes in renal tubular epithelial cells derived from a model of unilateral ureteral obstruction (45–48). Moreover, genetic and pharmacological inhibition of P53 has been shown to modulate kidney repair after AKI. Yang et al (49). showed that acute inhibition of P53 by a single dose of PIF on 14 d after renal ischemic injury ameliorated the development of renal fibrosis. Homogeneously, Ying et al. (50) reported that targeted deletion of p53 in proximal tubules prevents interstitial fibrosis in acute renal ischemia injury. In this study, firstly, our results showed that P53 and inflammatory factors as well as fibrosis factor levels dramatically increased at different times in endotoxin-induced AKI. Indeed, overexpression of AQP1 in septic AKI or inhibition of P53 via PIF reduces the inflammatory response while alleviating the level of fibrosis. Therefore, the decrease of AQP1 expression, up-regulation of P53 and inflammatory factors expression may be the main cause of renal fibrosis in septic AKI. However, Dagher (51) et al. showed that in a rat model of ischemic AKI, continuous injection of PIF for 2 d significantly alleviated renal damage, while continuous injection for 7 d significantly increased the pro-inflammatory function of macrophages and ultimately promoted renal fibrosis. This may be related to the type and pathological mechanism of AKI and the type of mouse and target cells.

AQP1, a transmembrane protein with molecular weight of 28kDa and a member of aquaporin family, plays an important role in the pathological process of septic AKI (5, 7). Rump and colleagues (52) found that aquaporin mainly regulate the migration of different immune cells of immune system in sepsis. Li (53) and Liu (7) confirmed that AQP1 promoted M2 phenotype polarization of RAW264.7 macrophages through PI3K or inhibition of P38 MAPK pathway after LPS stimulation, and alleviated renal inflammatory response and injury. In addition, the researchers showed that AQP1 protein dramatically increased first and then decreased in kidney tissue and serum, while AQP1 mRNA decreased significantly (7, 53). It was consistent with the results of our study that AQP1 protein have a trend of first increasing and then decreasing in renal tissue and serum in AKI with sepsis, while AQP1 mRNA decreased significantly. However, AQP1 protein expression was significantly reduced in LPS-stimulated HK-2 cells, without a trend of first increasing and then decreasing. There are several explanations for this phenomenon: 1) In the acute stage of septic AKI (12 h), AQP1 protein was dramatically elevated as a stress protein to play a protective role in the kidney. We confirmed that silencing AQP1 expression aggravated LPS-induced renal and cellular injury, mainly including up-regulating P53 expression and promoting inflammatory response, apoptosis and fibrosis levels. Wang et al. (54) demonstrated that AQP1 knockout will be accelerated LPS-induced apoptosis and promote the secretion of inflammatory factors, while AQP1 overexpression could reverse the damage of HK-2. This finding supports the results of our *in vitro* experiments. 2) After LPS-induced renal injury, the renal tissue was severely damaged and the microcirculation capillaries were congested, and the destruction of red blood cells resulted in the release of AQP1 on the erythrocyte membrane into the tissue space. AQP1 was first discovered and successfully demonstrated on mammalian red blood cells (55). Thus, AQP1 on the cell membrane is released into the blood when red blood cells are broken in large numbers, leading to a significantly increased. 3) The transiently increased of AQP1 may be caused by the change of AQP1 expression in macrophages. A study of leukocytes in the blood of ICU patients with sepsis showed that AQP1 was induced to be upregulated in leukocytes of ICU patients with acquired sepsis, resulting in higher AQP1 expression in leukocytes of patients with septic shock (56). 4) *In vitro* experiment time is single. LPS-cultured HK-2 cells only had a time point, lacking dynamic changes of AQP1 expression were observed.

In conclusion, transcriptional sequencing analysis showed that AQP1 expression was remarkably decreased and P53 expression was greatly increased in renal tissues and peripheral blood samples from experimental AKI models or septic AKI patients, suggesting that both AQP1 and P53 are involved in the development of septic AKI. In addition, we observed for the first that the expressions of AQP1 and P53 in kidney tissues and serum of rats significantly increased first and then decreased at different periods of LPS-induced AKI, which may be a novel marker for the early diagnosis of septic AKI. Our *in vivo* and *in vitro* experiments demonstrated that the silencing of AQP1 promoted inflammatory responses, increased apoptosis and interstitial fibrosis through up-regulation of P53 expression, which will be aggravated LPS-induced renal injury. AQP1 plays a protective role in modulating AKI and overexpression of AQP1 attenuated inflammation, apoptosis and fibrosis levels by inhibiting P53 expression in sepsis-induced AKI. Therefore, these results indicated that AQP1 is an important mediator of septic AKI and that pharmacological targeting of AQP1 mediated P53 signaling may provide a novel approach for the prevention or treatment of septic AKI.

## Data Availability

The datasets presented in this study can be found in GEO Repository, accession numbers: GSE186822, GSE220812, GSE667401, GSE248078 and GSE232404, and are included in the article/**Supplementary Material**, further inquiries can be directed to the corresponding author/s

## Supplementary material

Supplementary Figure 1 The CLP-induced AKI model was constructed. **(A, B)** Scr and BUN levels in serum. **(C)** pathology injury of the renal tissue of rats with CLP-induced AKI. Photomicrographs of HE stained kidney sections (200×). **(E)** Quantitative plot of renal tubular injury. **P <0.05, **P <0.01, ***P <0.001, ****P <0.0001, or ns p >0.05.*

Supplementary Figure 2 Levels of AQP1, P53, P21, inflammatory response and fibrosis were evaluated during CLP-induced AKI. **(A, B)** Expression of AQP1, P53 and P21 proteins in renal tissue and serum. **(C)** Levels of AQP1 mRNA, P53 mRNA and P21 mRNA in renal tissue in CLP-induced AKI were detected by RT-qPCR. **(D, E)** Expression of inflammatory factor (IL-1β, pNF-kB, TNF-α, IL-6) in rat kidney tissue and serum at 24 h of CLP treatment. **(F)** The kidney tissues of rats were prepared by immunohistochemistry to observe the number of inflammatory cell infiltration in renal tissue in CLP-induced AKI. **(G)** Quantitative graph of inflammatory cell infiltration in renal tissue. **(H)** The kidney tissues from rats were prepared by Masson staining in CLP-induced AKI (200x). **(I)** Quantitative plot of the level of interstitial fibrosis in renal tissue. **P <0.05, **P <0.01, ***P <0.001, ****P <0.0001, or ns p >0.05.*

Supplementary Figure 3 Identification and bioinformatic analysis of differentially expressed genes from GSE232404. **(A, B)** Expression of AQP1 and P53 in whole blood of patients with sepsis-induced AKI. **(C)** Heatmap of GSE232404. ***P <0.01, ***P <0.001.*
