# Supplementary figures and images for: Aquaporin 1 is renoprotective in septic acute kidney injury by attenuating inflammation, apoptosis and fibrosis through inhibition of P53 expression
Source: Front Immunol. 2024 Aug 22;15:1443108. doi: 10.3389/fimmu.2024.1443108 (PMC11374652; doi:10.3389/fimmu.2024.1443108)

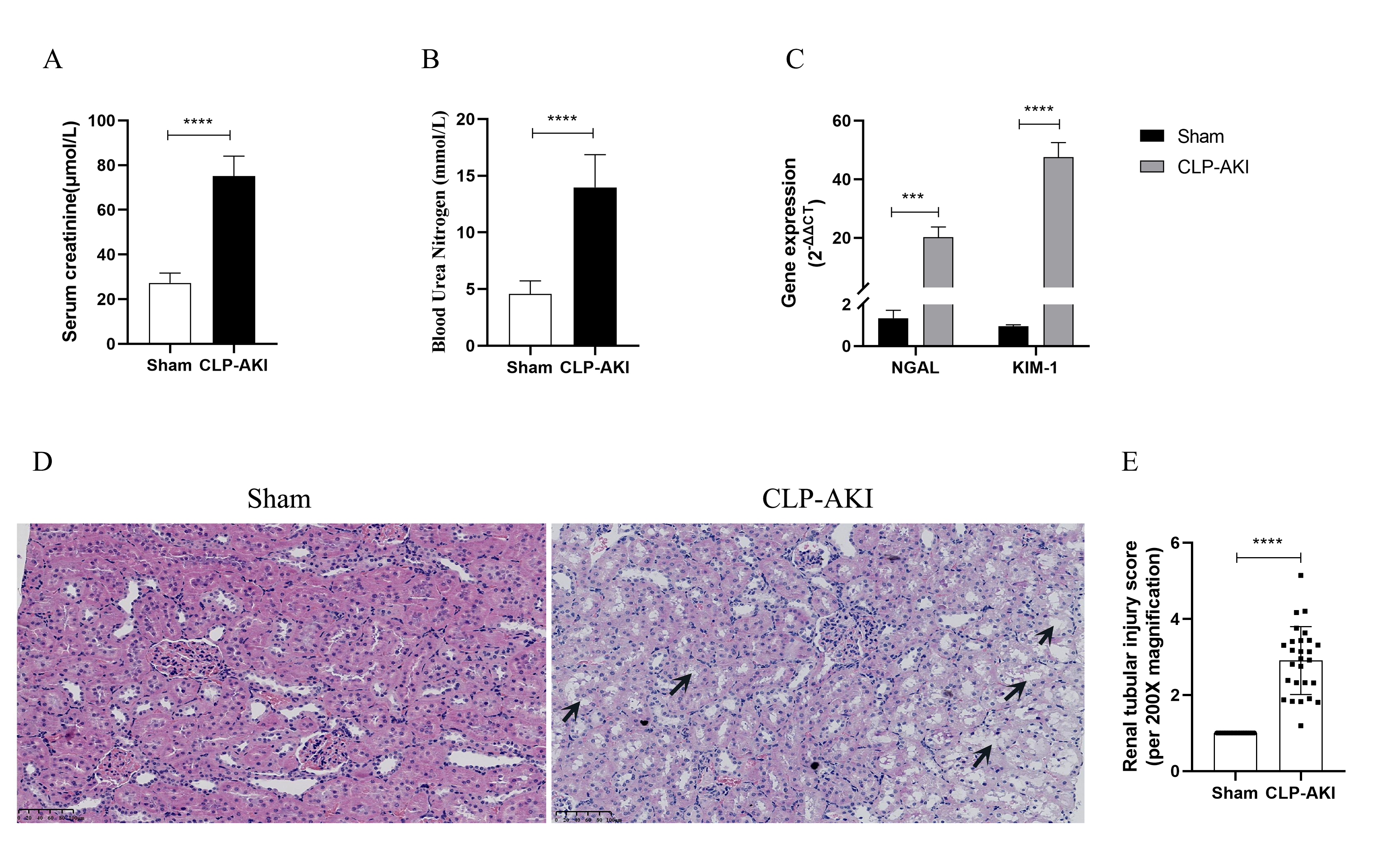

Supplement: Supplementary Figure 1 — The CLP-induced AKI model was constructed. (A, B) Scr and BUN levels in serum. (C) pathology injury of the renal tissue of rats with CLP-induced AKI. Photomicrographs of HE stained kidney sections (200×). (E) Quantitative plot of renal tubular injury. *P <0.05, **P <0.01, ***P <0.001, ****P <0.0001, or ns p >0.05. [file Image1.tif]

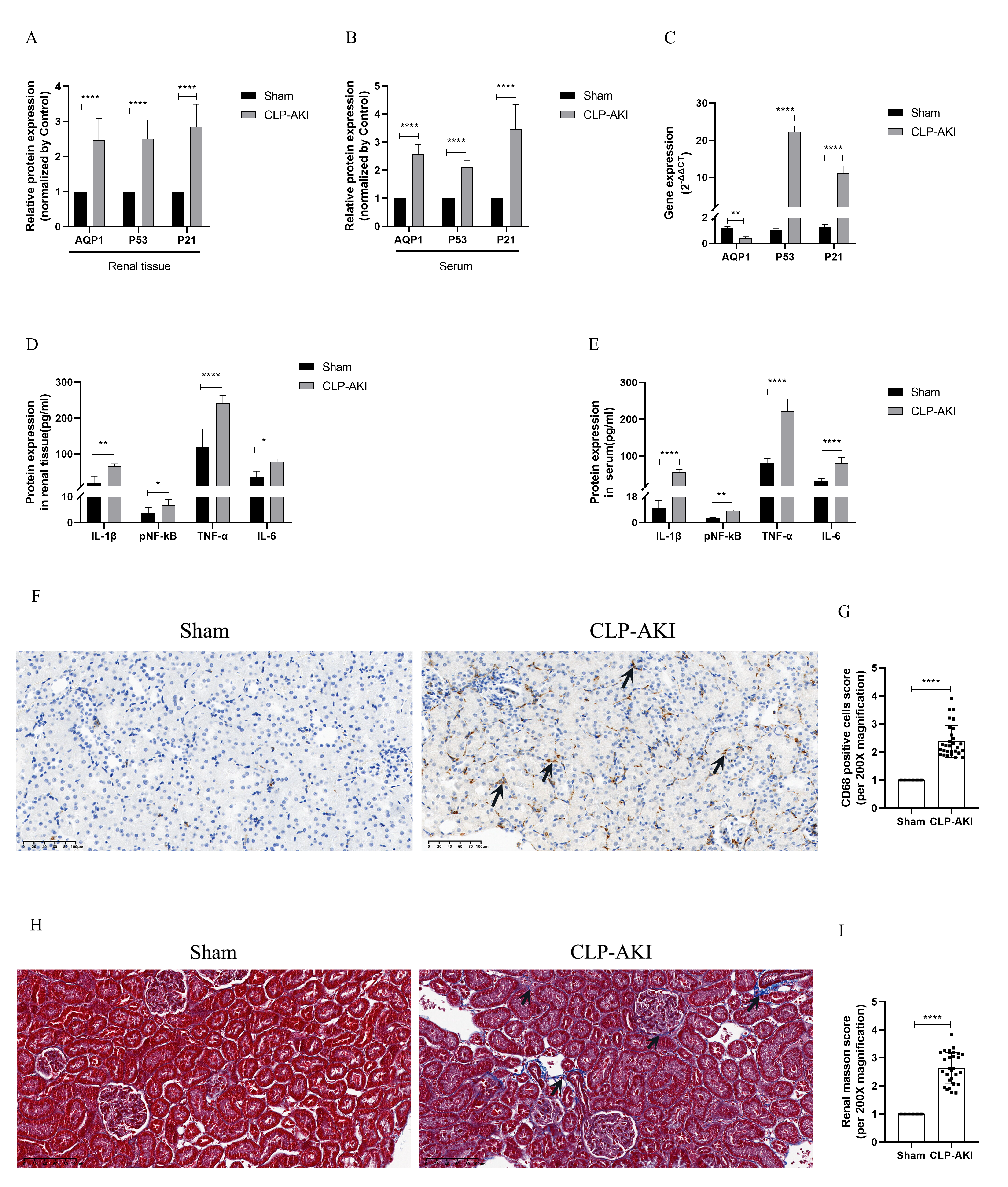

Supplement: Supplementary Figure 2 — Levels of AQP1, P53, P21, inflammatory response and fibrosis were evaluated during CLP-induced AKI. (A, B) Expression of AQP1, P53 and P21 proteins in renal tissue and serum. (C) Levels of AQP1 mRNA, P53 mRNA and P21 mRNA in renal tissue in CLP-induced AKI were detected by RT-qPCR. (D, E) Expression of inflammatory factor (IL-1β, pNF-kB, TNF-α, IL-6) in rat kidney tissue and serum at 24 h of CLP treatment. (F) The kidney tissues of rats were prepared by immunohistochemistry to observe the number of inflammatory cell infiltration in renal tissue in CLP-induced AKI. (G) Quantitative graph of inflammatory cell infiltration in renal tissue. (H) The kidney tissues from rats were prepared by Masson staining in CLP-induced AKI (200x). (I) Quantitative plot of the level of interstitial fibrosis in renal tissue. *P <0.05, **P <0.01, ***P <0.001, ****P <0.0001, or ns p >0.05. [file Image2.tif]
